# Molecular histopathology of matrix proteins through autofluorescence super-resolution microscopy

**DOI:** 10.1038/s41598-024-61178-0

**Published:** 2024-05-08

**Authors:** Biswajoy Ghosh, Jyotirmoy Chatterjee, Ranjan Rashmi Paul, Sebastian Acuña, Pooja Lahiri, Mousumi Pal, Pabitra Mitra, Krishna Agarwal

**Affiliations:** 1 Indian Institute of Technology Kharagpur, Kharagpur, West Bengal 721302 India; 2 Guru Nanak Institute of Dental Sciences and Research, Kolkata, West Bengal 700114 India; 3 UiT - The Arctic University of Norway, 9019 Tromsø, Norway

**Keywords:** Imaging, Microscopy, Biological techniques, Medical research, Oncology

## Abstract

Extracellular matrix diseases like fibrosis are elusive to diagnose early on, to avoid complete loss of organ function or even cancer progression, making early diagnosis crucial. Imaging the matrix densities of proteins like collagen in fixed tissue sections with suitable stains and labels is a standard for diagnosis and staging. However, fine changes in matrix density are difficult to realize by conventional histological staining and microscopy as the matrix fibrils are finer than the resolving capacity of these microscopes. The dyes further blur the outline of the matrix and add a background that bottlenecks high-precision early diagnosis of matrix diseases. Here we demonstrate the multiple signal classification method-MUSICAL-otherwise a computational super-resolution microscopy technique to precisely estimate matrix density in fixed tissue sections using fibril autofluorescence with image stacks acquired on a conventional epifluorescence microscope. We validated the diagnostic and staging performance of the method in extracted collagen fibrils, mouse skin during repair, and pre-cancers in human oral mucosa. The method enables early high-precision label-free diagnosis of matrix-associated fibrotic diseases without needing additional infrastructure or rigorous clinical training.

## Introduction

Several diseases having high clinical burdens are associated with changes in matrix proteins. Fibrosis is one such pathology of collagen that is caused by chronic diseases like systemic sclerosis, glomerulonephritis, interstitial lung disease, non-alcoholic steatohepatitis (NASH), Crohn’s disease, and heart disease^1^. Fibrosis leads to organ damage of several vital organs like the kidney, liver, lungs, and heart and influences cancer metastasis in the oral cavity, liver, and lungs^2^. Fibrosis is also of significance in wound repair and regenerative medicine^3,4^. While controlled fibrosis is a natural outcome of wound healing, in several cases like chronic diseases, excessive fibrosis in the wound jeopardizes the function of the organs^5^. Matrix proteins provide structural integrity to tissues, and changes in these proteins by density, distribution, or structure impact function. The realization of tissue matrix abnormalities can, therefore, be good indicators of pathological manifestation^6^. Visualizing spatial protein density across different tissue zones through optical microscopy is therefore a key to histopathology examinations.

Conventional histopathology often employs staining specific matrix proteins using targeted immuno-staining techniques like immunofluorescence and immunohistochemistry. Although they have high molecular specificity, they suffers from constraints like (i) the difference in spatial location of the staining molecules and the actual molecule^7–11^ compromises the structural information; (ii) irregularity in the number of fluorescent probes per binding site upon indirect immunostaining compromises density information^7^; and (iii) underexposed epitopes that are unavailable for probe binding causes incomplete labelling^12^. Other histochemical stains like van Gieson are regularly used for staining collagen, but they have lower specificity and sensitivity compared to immune-labeling methods^13^. Electron microscopy (EM) and atomic force microscopy (AFM) allow label-free imaging of matrix proteins^14^ and provide an additional advantage of resolution ($\sim$ 2 nm) unmatched by the conventional optical microscopes even with very high magnification and numerical aperture (200 nm). However, the extremely small spatial and axial field provides a limited perspective of the tissue making conclusive findings obscure.

A long-sought ideal is to identify early modifications in the matrix density label-free with high-resolution optical microscopy. The occurrence of several autofluorescent molecules in biological cells and tissues^15–17^ has prompted their use for label-free investigations using conventional optical microscopes in several niche areas like cancer diagnosis^18^ and regenerative medicine^19^. Autofluorescence seems to be ideal for super-resolution imaging^8,10^ as it is source-emitted (better localization and fidelity in distribution); therefore, it overcomes labeling limitations. Fluorescence imaging applications like multi-photon microscopy and second harmonic generation imaging also use native optical properties of collagen for imaging them^20,21^. However, the autofluorescence methods for tissue collagen and keratin mentioned above are diffraction-limited which limits how well they can resolve small structures. On the other hand, super-resolution optical microscopy that surpasses the diffraction-limited resolution, or nanoscopy in short, for fluorescently labeled samples has come a long way^22,23^, even for tissue samples^24^. A recent study by Barlow et al. has shown the use of the Zeiss Airyscan (a 32-channel GaAsP photomultiplier tube array detector^25^) on top of multiphoton microscopy and second harmonic generation imaging can improve resolution up by a factor of 1.7$\times$^26^. However, the potential of nanoscopy beyond just the resolution aspect for autofluorescence is not explored^7^.

Optical nanoscopy methods see high resolution but lose the larger clinically relevant context. Studies that reported nanoscopy for autofluorescence signal did so for cellular and sub-cellular structures^8–10^. The challenge in translating such techniques to tissue is due to dense tissue matrix proteins such as collagen (connective tissue) and keratin (epithelial tissue) with highly variable local densities^7,12,27,28^. Single-molecule localization-based super-resolution methods like photoactivated localization microscopy (PALM), stochastic optical reconstruction microscopy (STORM), and point accumulation for imaging in nanoscale topography (PAINT) have strict requirements on the sample preparation and imaging. Thus, specialized probes and chemical interventions are mandatory, restricting the usage of a native property like autofluorescence. Nanoscopy methods like stimulated emission depletion (STED), ground state depletion microscopy (GSD), and reversible saturable optical fluorescence transition (RESOLFT) are feasible in principle for tissue super-resolution because of their point scanning mechanism. These methods have worked with labeled fluorescence in relatively sparse and intracellular targets^9,10^, but their application for imaging autofluorescence in dense structures is pending exploration. In addition to purely optical methods for super resolution, computational nanoscopy has gained popularity. This is primarily because computational methods do not need very sophisticated optical systems for achieving nanoscopy. Computational nanoscopy methods work post-acquisition and have fewer restrictions and less demanding sample preparation. Multiple signal classification algorithm (MUSICAL), is one such method that uses singular value decomposition over the entire image-stack and then attempts to separate signal from noise. The resolution gain comes from using the information of the noise against the expected point spread function (PSF) of the system. Comparatively, MUSICAL provides better results in terms of resolution and quality of reconstruction than other similar methods like super-resolution optical fluctuation imaging (SOFI)^29^, especially for noisy data and high-density structures. On the other hand, another such method super-resolution radial fluctuations (SRRF) is able to produce high-resolution images but also tends to distort structures making them narrower^30^ which is not the case with MUSICAL. A more extensive study has been made by Opstad et al^31^. It is to be noted that super resolution techniques are signal processing techniques and not data acquisition systems.

Another challenge is to include sufficient sample thickness for clinical relevance. The optical restrictions that most super-resolution methods impose, limit the usable thickness of the sample space for imaging to be as low as 500 nm (for a 100 $\times$ objective, 1.4 NA). Such restrictions, in essence, remove the information of prominent density variations otherwise seen in microscopic evaluation of histopathological samples of 4–5 $\upmu$m section thickness. Even if super-resolution methods achieve a wide field of view by stitching individual images, it does not integrate the depth information necessary to assess protein density variation and compare pathologies. Lastly, point-scanning-based super-resolution techniques require the microscope to be confocal and limit throughput^7^. In summary, nanoscopy for autofluorescence in tissues remains a challenge^7^, especially in a histopathologically relevant view field.

Here, we present the use of a computational nanoscopy method-multiple signal classification algorithm (MUSICAL)^29^ beyond its primary task of super-resolution but rather better density estimation. This allows for capturing density information of otherwise indiscernible dense matrix proteins in clinical tissue samples label-free and using just a conventional optical microscope. MUSICAL requires that fluctuations in fluorescence intensity are measured in the camera, irrespective of the mechanism underlying the fluctuations and the density of emitters. We adapted this feature in the large depth of field nanoscopy using a low magnification objective. A broad field of view is an added advantage of using a low magnification objective, enabling visualization across a larger portion of the tissue. The choice of optics and multiple signal classification algorithms coupled with the described advantages of autofluorescence makes MUSICAL-tissue autofluorescence (MUSI-tAF) ideal for imaging protein density variations. Further, the method’s ability to suppress out-of-focus background^29^ enables it to strictly visualize the density of a fixed tissue thickness across the objective’s depth of field. Thus, MUSI-tAF projects a ‘volumetric’ density variation, e.g., $\sim 323 \times 435 \times$1.2 $\upmu \textrm{m}^3$ (for a 0.80 NA objective) on a 2D super-resolved image plane.

**Figure 1:**
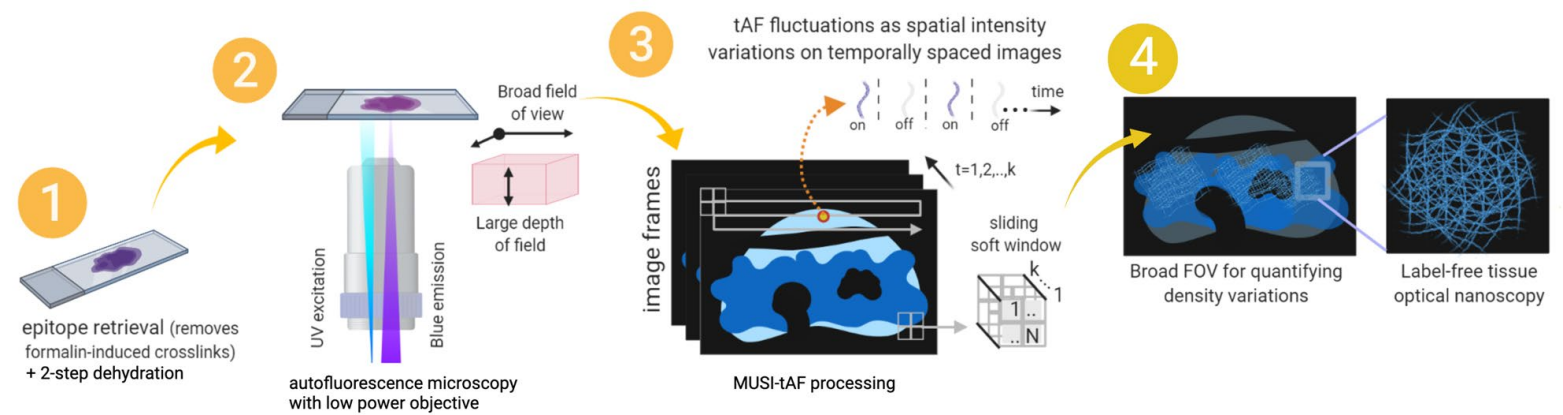
Schematic diagram for MUSI-tAF workflow. The FFPE tissue sections replete with autofluorescing matrix proteins are mounted on glass slides, deparaffinized and dehydrated. Epitope retrieval is performed to remove unwanted formalin-linked fluorescence. A low magnification epifluorescence microscope (20 $\times$, 0.80 NA objective) that allows a broad field of view (FOV) of $\sim 323 \times 435\, \upmu$m^2^ and a thicker depth of field (DOF) of $\sim 1.2 \upmu$m is used to acquire image stacks at excitation and emission wavelengths specific to matrix protein autofluorescence. The rapidly acquired image stacks are processed by the multiple signal classification algorithm to achieve super-resolution as previously described^29^. The MUSI-tAF image presents quantifiable density variations on a broad field for a better clinical context along with the nanoscopy of matrix collagen protein.

## Methods

### Ethical considerations

We performed the animal study as per the Institute Animal Ethical Committee (IIT Kharagpur, Kharagpur) regulations. Wound healing study in mice was done under approval # IE-1/JC-SMST/2.18. Animal studies for ANE-induced pathological fibrosis was done under approval # IE-7/JC-SMST/2.18. We confirm that that all experiments were performed in accordance with relevant guidelines and regulations, and the authors complied with the ARRIVE guidelines. Likewise, the human oral pre-cancer and cancer study was approved by the Institute Human Ethics Committee of the source Hospital (GNIDSR, Kolkata, India) under approval # GNIDSR/IEC/ECC/2015/06. The authors confirm that informed consent was taken from all the participants.

### Samples

We procured commercially available purified collagen-I derived from rat tail (Cat No. C7661, Sigma-Aldrich; A1048301, Thermo Fisher Scientific). We prepared 15 slides of rat tail collagen on microscope cover glasses. This was used as a starting sample to test MUSI-tAF to validate resolution improvement, comparison with other objectives, and comparing auto-florescence and immunolabelling. We obtained the human oral samples (oral cancer and precancer) after biopsy and clinicopathological validation from GNIDSR, Kolkata, India. Two independent oncopathologists evaluated the samples for staging using histopathology. N = 5 biopsied and formalin-fixed paraffin-embedded specimens were used per group (NOM, OSF, OSFD, OSCC, OLKP). Multiple ROIs (N $\ge$ 30) were collected from each group. For making scar tissues, we created skin full-thickness wounds ($\sim$ 4 mm diameter) on the dorsum of the mouse (male, age 2 months, Swiss albino, weight 30–40 g) and allowed them to heal for a month till a scar was distinctly visible. We extracted the scar by excision and fixed the samples for further processes. For preparing arecanut extract and for the creation of chemically induced fibrosis in mice, we followed the protocol described previously^32^. We extracted the fibrotic skin tissue at different time intervals by excision for further processing. We prepared the arecanut extract in-house using locally available arecanut. N = 4 animals were used in each group of arecanut fibrosis (Normal, F18, F30, F60, F180) and scar tissue. Multiple ROIs were collected from each group as well for statistical analysis. The overview of the numbers of samples, images and ROI’s used in the study is provided in the supplementary Table S1.

### Sample fixation and processing for MUSI-tAF

We submerged the tissue samples in 10% phosphate-buffered formalin for tissue fixation for 24 hours. We vacuum-embedded the fixed tissues in melted paraffin to obtain tissue blocks. We cut the formalin-fixed paraffin-embedded (FFPE) tissue into thin sections of 5 $\upmu$m and mounted them on glass slides coated with poly-L-lysine (0.1% w/v in water, Cat. No. P8920, Sigma Aldrich, Germany). We then removed the paraffin by incubating slides dipped in warm (60 ^∘^C) xylene for 30 min, followed by immersion of the slides in 100% ethanol for 5 min. We performed antigen retrieval on the sections using Tris-EDTA buffer (pH 9.0) using EZ-Retriever System V.2 (BioGenex) with the standard microwave protocol. We dehydrated the samples in graded alcohol (50% and 70%, 5 min each) up to 100%, 45 min followed by air-drying them overnight, and 60-min oven-baking at 45 ^∘^C before microscopy.

### Tissue staining

For immunostaining (IHC and IF), after antigen retrieval, we incubated the FFPE tissues with primary antibodies (monoclonal anti-collagen-I, Cat No. ab6308, Abcam; polyclonal anti-collagen-I, NB600-408, Novus Biologicals; monoclonal anti-Pan-keratin, No. 4545, Cell Signaling Technology) overnight at 4 ^∘^C with the factory recommended dilutions. We chose a red fluorescent probe (Alexa Fluor 594) for visualizing labeled collagen-I to measure colocalization with collagen autofluorescence in the blue emission spectra. For IHC, we used horseradish peroxidase-conjugated secondary antibodies using the chromogen 3,3’diaminobenzidine (DAB) for pan-keratin staining. We performed nuclear counterstaining using hematoxylin in all the IHC slides. For clinical validation and staging of precancer/cancer, we used the hematoxylin and eosin (HE) stained slides. For staining the subepithelial connective tissue, we performed the van Giesons stain (VG) (differentially stains collagen and muscle fibrils in red and other components in yellow) with hematoxylin nuclear counterstaining.

### Imaging parameters

We imaged the tissue sections with an inverted wide-field epifluorescence microscope (Axio Observer.Z1, Zeiss, Germany)) under a 20 $\times$ objective (NA = 0.80, Zeiss, Germany). We captured about 500 frames with an exposure time of 20–30 ms and a frame rate of 14 fps to acquire the emission fluctuations from the autofluorescent proteins. The CCD camera (AxioCam MRm, Zeiss, Germany) had a square pixel size of 6.45 $\upmu$m. We saved the image frames in a non-compressed OME-TIFF format for MUSICAL. We illuminated the samples using a 120 W mercury plasma arc-discharge lamp (X-Cite Series 120 Q, Excelitas Technologies, NYC, USA), having excitation/emission spectra filter cubes (Chroma Technology, Bellows Falls, VT). For imaging at 50 ms exposure time, 100% of the lamp power was used. For detecting collagen autofluorescence, we used excitation (ex) and emission (em) peak of $\lambda _{ex}$ = 359 nm and $\lambda _{em}$ = 461 nm, and for Alexa Fluor 594 labeled fluorescence, we used $\lambda _{ex}$ = 592 nm, $\lambda _{em}$ = 614 nm. The spatial dimension of microscope images-1388$\times$1040 pixels translates to a field of view (FOV) of 448$\times$335 $\upmu$m^2^ in the physical space. For a wavelength ($\lambda$) = 460 nm, the refractive index of the medium ($\mu _{air}$) = 1, NA = 0.80, magnification = 20$\times$, and pixel size (*e*) = 6.45  $\upmu$m, the images had a depth of field (DOF) of $\sim \, 1.2\,\upmu$m as per the following equation:

1 $$\begin{aligned} DOF=\frac{\lambda \cdot \mu }{NA^2} + \frac{\mu }{M \cdot NA}e \end{aligned}$$

The diffraction-limited (DL) image with a high magnification (1.4 NA, 100$\times$) objective has a much smaller FOV (90 $\times$ 67 $\upmu$m^2^) and DOF (0.28 $\upmu$m) compared to the low magnification DL image.

### Monolayer deposition of rat tail collagen

To compare MUSI-tAF results with SEM, we required a monolayer of collagen. We used rat tail collagen-I for this purpose (A1048301, Thermo Fisher Scientific). The collagen was mixed with 10$\times$ PBS and sodium hydroxide as per manufacturers’ instructions to prepare a final concentration of 0.2 mg/ml. 200 $\upmu$l of the collagen solution was allowed to dry in coverslips for 24 hours. After the drying stage, the collagen was washed 5$\times$ in distilled water to remove any excess salt deposits that may interfere with imaging. The collagen was allowed to air dry at room temperature for 48 hours before imaging. Ideally, the sample is a monolayer but we expect several areas to be 3-5 collagen fibril thickness.

### MUSI-tAF

We used the Matlab source code developed by Krishna Agarwal for generating MUSICAL results. The codes are available at the link (https://sites.google.com/site/uthkrishth/musical). We used a sub-pixelation of 10, i.e., the 10$\times$10 MUSICAL pixels correspond to one physical camera pixel. As suggested previously, we chose the threshold for each image heuristically close to the knee of singular value plots. The parameter $\alpha$ was assigned the default value of 4.

MUSI-tAF, a multi-frame high-density super-resolution method, uses an indicator function ($i_\mathrm{MUSI-tAF}$), which is calculated based on microscope optics-driven modeling of signal (*s*) and noise (*n*). This modeling in turn involves statistical separation of optical mapping (technically called point spread function and abbreviated as PSF) of sample structure from noise using singular value decomposition as explained in the following. The value of the indicator function at a point *p* in the sample region indicates the presence of a fluorescence emitter as:

2 $$\begin{aligned} i_\mathrm{MUSI-tAF}(p)=\Big (\frac{signal}{noise}\Big )^\alpha \end{aligned}$$

The algorithm calculates the indicator function map sequentially as a square window of size N (matching the spatial spread of the PSF) slides across the image’s spatial dimension and stitches them later. The scale factor $\alpha$ controls the contrast in each sliding window and contributes to smoothness across the windows during the stitching operation. For our results, we used $\alpha = 4$. For further reading on the mathematical derivations, more insight into the variables, and comparison with other optical nanoscopy methods, the readers are referred to^29^.

### Image and statistical analyses

Co-localization of labeled collagen fluorescence vs. collagen autofluorescence was performed using MUSICAL for both autofluorescence and labeled fluorescence of Collagen-I and measured the structural similarity using SSIM (structural similarity index) in MATLAB R2019a. We performed the DAB and hematoxylin color deconvolution in ImageJ using the plugin developed by Gabriel Landini available at https://imagej.net/Colour_Deconvolution. ImageJ line tool was used to determine fibril-thickness from MUSI-tAF images with a spatial field of $5\,\times \,5 \, \upmu \textrm{m}^2$ to clearly visualize fibrils. A total of N = 300 fibrils per group was used to generate a histogram plotted in GraphPad Prism 8. For the measurement of intensity and variance in oral pre-cancer layers, random patches were generated in specific regions/layers of the tissue to be measured. Intensity ratios were measured by generating the combinations of intensities between the two participant layers. For every paired comparison, a paired 2-tailed t-test was used to assess the *p*-value. For multi-group comparisons, ANOVA with Tukey’s modification was used and the *p*-value was measured. For multi-scale analysis for every window size, pixel patches of 300$\times$300 were used. The measurements of keratin from stained OLKP samples, after color deconvolution, the DAB component was inverted and converted to 8-bit grayscale format before generating patches and quantifying the stain uptake.

### Ultra-structure imaging

For SEM, the samples were coated with gold-platinum alloy mist and imaged under SEM (MERLIN, Zeiss, Germany) up to 200,000$\times$ EM magnification to image the typical collagen fibril structures.

### Density measurement of matrix proteins

MUSI-tAF images display fibrous structure of the matrix proteins by improving resolution and reducing out-of-focus lights. To measure the density, a 100$\times$100 ROI is selected and the mean intensity is considered as the absolute density for the ROI. Several ROIs (at least 25) are selected for statistical analysis in each category. For clinical samples, in order to avoid image to image variations, relative densities are used as a metric instead of absolute density. For example, ratio of sub-epithelium (SE) to epithelium (E) denotes the density ratio of matrix proteins in sub epithelium relative to that of epithelium is measured. Similarly, ratios between different layers and sub-layers have been used to determine different clinically relevant metrics. All the measurements were done in Fiji platform. Details and clinical relevance of these metrics are provided in the supplementary Table S2.

**Figure 2:**
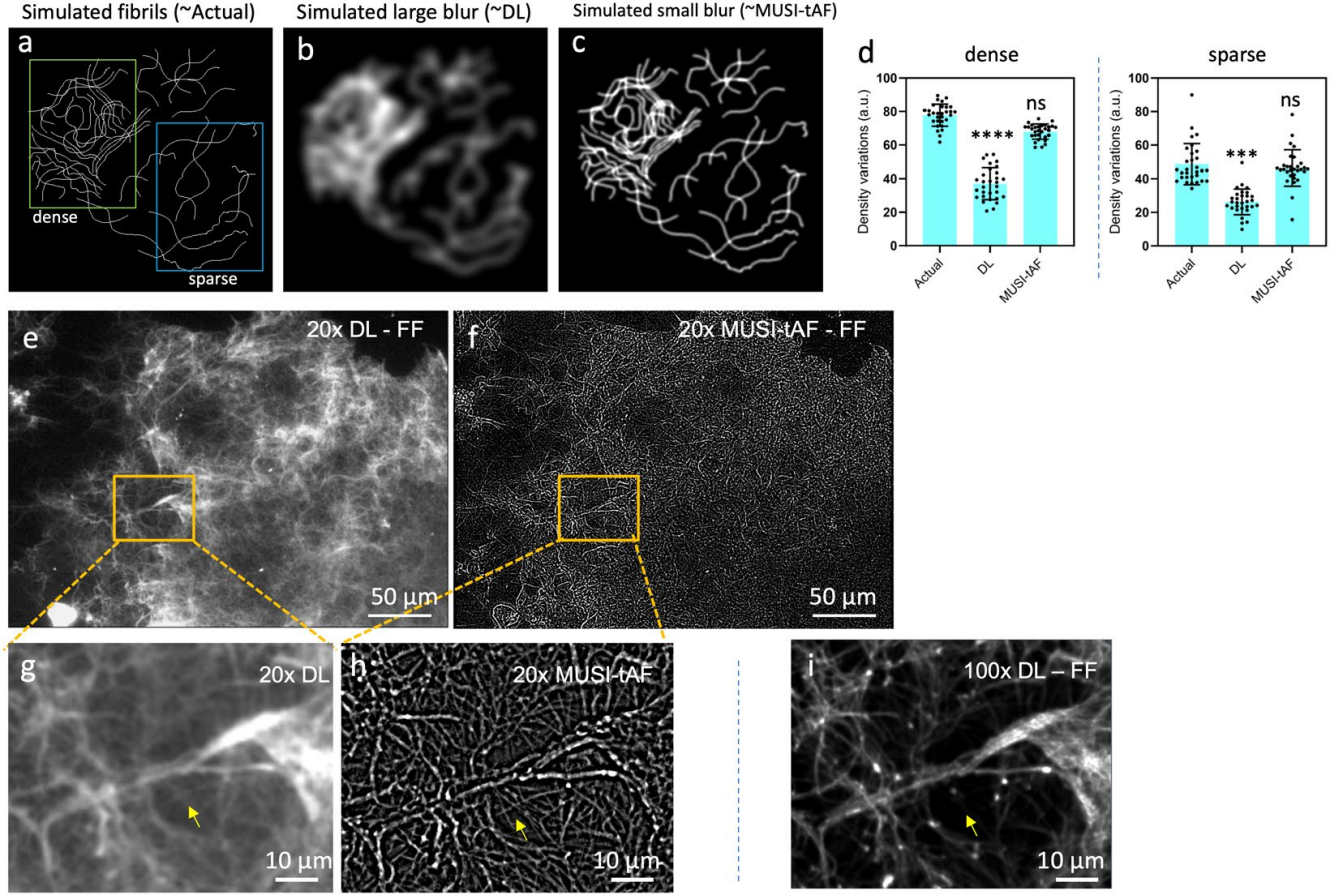
MUSI-tAF provides a better estimate of density variation of dense collagen. We simulated dense fibrils to represent (**a**) actual structures, (**b**) diffraction-limited (DL) image, and (**c**) MUSI-tAF by introducing a blur function. (**d**) Quantification of the density variation in both dense and sparse regions showed that MUSI-tAF is closer to the actual fibrils. N = 30 per group, all plots are represented as $\mu$ ± SD, ns (not significant), ***p $\le$ 0.001, ****p $\le$ 0.0001. For statistical analysis, we used one-way ANOVA (95% confidence interval). The effect was realized in full-field (FF) images of rat tail collagen-I with both dense and sparse regions under (**e**) diffraction-limited and corresponding (**f**) MUSI-tAF. A zoomed-in region of (**g**) diffraction-limited and (**h**) MUSI-tAF shows resolution enhancement in closely spaced fibrils (yellow arrows). Comparison of (**h**) with (**i**) full-field 100$\times$ diffraction-limited image shows MUSI-tAF images fibrils from a thicker section of the tissue as it is rendered from a low magnification objective with larger DOF, while such regions are rejected in 100 $\times$ images with a smaller DOF (yellow arrows).

## Results

### MUSI-tAF approach for optical nanoscopy of tissue autofluorescence

Figure 1 summarizes the steps involved in generating a MUSI-tAF image. Collagen and keratin molecules need excitation in the UV region (320–410 nm) to emit autofluorescence in the blue region (400–510 nm)^15,16,20,33^ of the electromagnetic spectra. Thus, we used suitable microscope filter cubes (Supplementary Fig. S1) to collect the desired autofluorescence. The image frames captured on a low magnification objective (20 $\times$, 0.80 NA,) accumulated the desired photokinetic fluctuations as spatial variations in fluorescence intensities across the frames. We pre-process the samples to minimize interference from moisture and formaldehyde (fixative)-associated fluorescence^34^ (Supplementary Fig. S2). For MUSI-tAF we use microscopy image stacks (k $\approx$ 500) with a frame rate of 14 fps with an exposure time of 15–100 ms. Details of the algorithm are presented in the Methods section. Benchmarking of the structural fidelity of MUSI-tAF and its comparison with SEM is presented in Supplementary Fig. S3. The validation of the structural reliability of autofluorescence over labeling is presented in Supplementary Fig. S4. A comparison of label-free autofluorescence and labeled fluorescence is presented in Supplementary Fig. S5 and Supplementary Notes 1–4.

### MUSI-tAF provides a better estimate of collagen density variations

Here, we show that in addition to resolving nanoscale features, super-resolution imaging also has a role to play in more accurate density representation at macroscopic scales (across hundreds of microns). We illustrate this aspect using a simulated example first. we simulated fibrils along with different levels of blur (Fig. 2a–c). A large blur represents diffraction-limited imaging (Fig. 2b) and a small blur represents the corresponding super-resolved image (Fig. 2c), analogous to the MUSI-tAF image. The super-resolved image provides a closer representation of density variation in the actual fibrils in comparison to the diffraction-limited image (Fig. 2d). It is thus expected that MUSI-tAF imaging also presents the advantage of super-resolution for more accurate density representation at macroscopic scales. In particular, an observation analogous to Fig. 2b,c is seen in the experimental diffraction-limited image (Fig. 2e) and MUSI-tAF image (Fig. 2f) of rat tail collagen fibrils. The high-intensity regions in the diffraction-limited images present a high density of fibrils in the super-resolved images, which translates to better density estimation. This aspect of improved macroscale representation of density, combined with clinically relevant large FOVs makes MUSI-tAF reconstructions valuable for translation to clinical studies. In addition to the above impact of super-resolution, MUSI-tAF also contributes to better density estimation along the depth of the tissue by rejecting out-of-focus signals (Supplementary Fig. S6). Lastly, since clinical context requires a sufficient thickness of imaging, low magnification MUSI-tAF’s ability to image larger depth of focus as compared to corresponding high magnification diffraction-limited image (also shown in Supplementary Fig. S5) is an added advantage for clinical translation. Thus, as a consequence of MUSI-tAF, we are on one hand able to super-resolve nanostructures from low magnification objectives with better density quantification (Fig. 2g,h) than diffraction-limited counterpart (Fig. 2g,h); on the other hand, as MUSI-tAF is essentially derived from a low magnification objective, it images collagen from a thicker sample depth compared to a 100$\times$, 1.4 NA objective (Fig. 2i, see yellow arrows). However, MUSI-tAF can resolve structures only up to a certain degree of density. In the case of highly condensed bundles containing hundreds of fibers packed tightly, it is beyond the resolving capacity of MUSI-tAF. For example, the bright bundle seen in the center of Fig. 2h, is highly condensed and the individual finer fibers are not visible.

**Figure 3:**
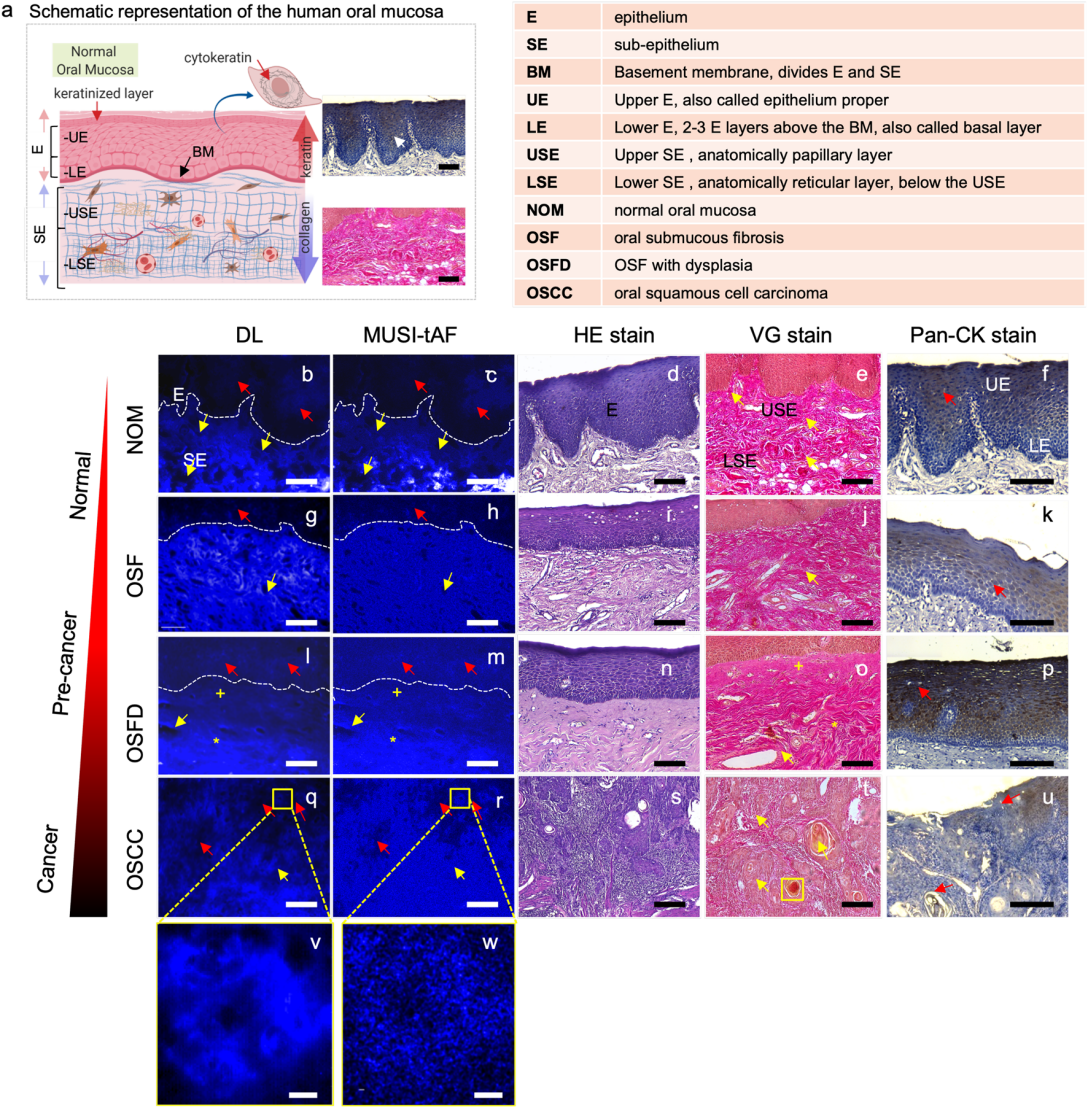
**MUSI-tAF for imaging pre-cancer progression in the human oral mucosa.** (**a**) The schematic representation of the human oral mucosa section showing the keratin-rich epithelium and collagen-rich sub-epithelium. Corresponding diffraction-limited (DL) image, MUSI-tAF, hematoxylin and eosin (HE) staining as the clinical gold standard, van Gieson (VG) staining for collagen in the sub-epithelium, and pan-keratin (pan-CK) immunostaining for keratin in the epithelium in (**b**–**f**) normal oral mucosa (NOM), (**g**–**k**) oral submucous fibrosis (OSF), (**l**–**p**) OSF with dysplasia (OSFD), (**q**–**u**) oral squamous cell carcinoma (OSCC), (**v**,**w**) insets of DL and MUSI-tAF of the oral carcinoma (OSCC) showing pearl like islands, which are markers of metastatic progression (see the yellow box in the VG stain figure **t**). In the pearl like islands in the DL image (**v**), the details are averaged out or saturated. While the MUSI-tAF image (**w**), clearly discerns the local density variations of the fibrous matrix proteins making intensity measurements quantitative. We have used three coloured arrows to compare MUSI-tAF with other clinically relevant images. The yellow arrows and markers (+,*) indicate sub-epithelial changes in MUSI-tAF corroborative with VG stain images that stains collagen (**e**,**j**,**o**,**t**); the red arrows mark ground truth matching epithelial changes in Pan-CK stain images. DL and MUSI-tAF images are region matched, while the other images are the pathological images taken from the corresponding samples, but are not region matched. *Scale bars*: (**b**–**u**) 50 $\upmu$m *and* (**v**,**w**) 5 $\upmu$m.

### MUSI-tAF aids imaging and detection of pre-cancer stages

Oral squamous cell carcinoma is a cancer of the mouth and it progresses through pre-cancer stages which when detected early can be useful for early clinical intervention. A schematic representation of the oral mucosa is presented in Fig. 3a along with a list of relevant abbreviations. The oral mucosa (Fig. 3a) has (i) a keratin-rich epithelium layer and (ii) a collagen-rich sub-epithelium just beneath the epithelium layer. In oral tissues, collagen and keratin are exclusively present in the sub-epithelium^16^ and the epithelium^35^, respectively. Since these two molecules overlap spectrally in autofluorescence^16,33^ but are anatomically differently localized with exclusivity, MUSI-tAF can image them simultaneously. We used MUSI-tAF to image four pre-cancer stages—(Fig. 3b–f) normal oral mucosa (NOM), (Fig. 3g–k) oral submucous fibrosis (OSF—benign pre-cancer), (Fig. 3l–p) OSF with dysplasia (OSFD—the high potential of malignancy), and (Fig. 3q–u) oral squamous cell carcinoma (OSCC—cancer). The stage-wise clinical diagnosis was performed by pathologists using the HE-stained images (Fig. 3d,i,n,s) (Supplementary Fig. S7). We employed appropriate histopathological and molecular imaging standards to validate the spatial abundance of the collagen (Fig. 3e,j,o,t) and keratin (Fig. 3f,k,p,u) proteins in the epithelium and sub-epithelium. Two independent oral oncopathologists supervised and confirmed all validations and marked the distinction of epithelium and sub-epithelium in the DL and MUSI-tAF images. The difference between the DL and MUSI-tAF can be seen clearly from the oral cancer insets shown in the Fig. 3v,w.

Collagen distributions in the sub-epithelium in MUSI-tAF images (Fig. 3c,h,m,r) matched with the corresponding VG stained sections (collagen-red color) (Fig. 3e,j,o,t) in all stages of oral pre-cancer. We used the VG stain to image collagen density variations as it stains all collagen types. The sub-epithelial tissues that are well-differentiated with distinct papillary (upper layer) and reticular (lower layer) sub-epithelium in NOM (Fig. 3c,e) showed homogeneous distribution in OSF (Fig. 3h,j). In OSFD, the reticular sub-epithelium showed high density compared to the papillary tissue with peri-vascular fibrosis (Fig. 3m,o). Due to the disruption of the sub-epithelial integrity in OSCC, the epithelial islands were seen among the sub-epithelium (Fig. 3r,t).

**Figure 4:**
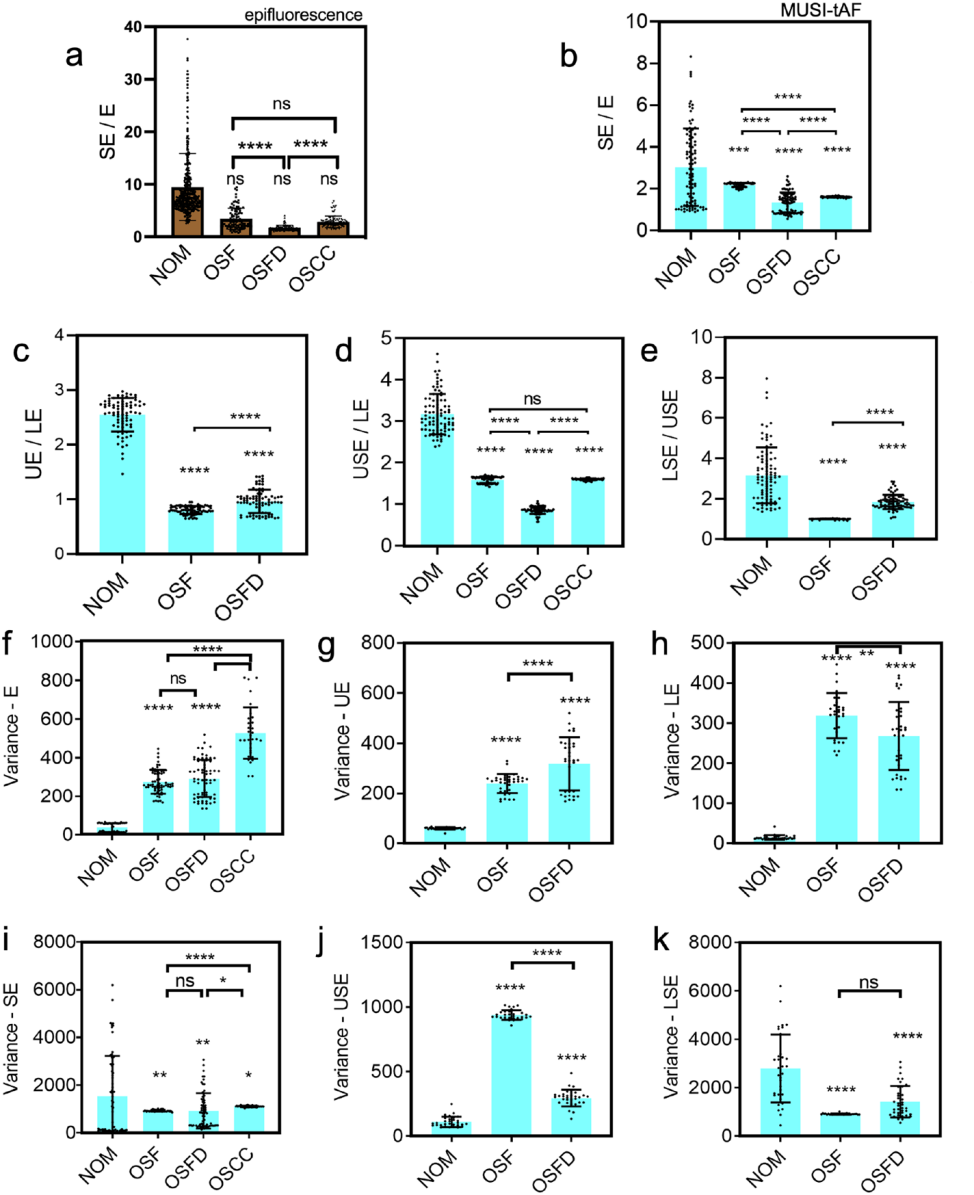
Quantification of broad-field MUSI-tAF features in delineating human oral pre-cancer stages. Ratio of (**a**) epifluorescence intensity and (**b**–**e**) MUSI-tAF intensity between the layers of epithelium and sub-epithelium to delineate the stages-NOM, OSF, OSFD, and OSCC. The used abbreviations can be found in Fig. 3. N = 60–100 per group (patient samples = 5 $\times$ ROI = 20). (**f**–**k**) Variance quantification of different layers and sub-layers of epithelium and sub-epithelium to delineate the oral pre-cancer stages. In OSCC tissues, the epithelium and sub-epithelium cannot be differentiated due to metastasis and hence were not included in (**b**,**d**,**f**,**g**,**i**,**j**). N = 30–60 per group (patient samples = 5 $\times$ ROI = 10) , All plots are represented as $\mu$ ± SD, ns (not significant) *p* > 0.05, *p $\le$ 0.05, **p $\le$ 0.01, ***p $\le$ 0.001, ****p $\le$ 0.0001. For statistical analysis, we used one-way ANOVA (95% confidence interval) followed by Tukey’s post hoc test.

We found spatial match between epithelial MUSI-tAF (Fig. 3c,h,m,r) and keratin-specific immuno-staining (keratin-brown color) (Fig. 3f,k,p,u) for all four stages. The normal stratified oral epithelium has high keratin expressing epithelium proper (upper epithelium) and low keratin expressing basal and supra-basal (lower epithelium) layers (Fig. 3c,f). However, keratin expression gradually increases and becomes less distinct between two epithelial zones in OSF (Fig. 3h,j) and OSFD (Fig. 3m,p). In cancer, the epithelial islands that invaded the sub-epithelium show lower expression of keratin compared to OSFD (Fig. 3r,u).

For quantification, we divided the epithelium (E) and sub-epithelium (SE) into two distinct zones (Fig. 3a, refer to the list of abbreviations). We annotated the tissue layers of epithelium proper as the upper epithelium (UE), basal/supra-basal epithelium as the lower epithelium (LE), papillary sub-epithelium as the upper sub-epithelium (USE), and reticular sub-epithelium as the lower sub-epithelium (LSE) in MUSI-tAF images. Interestingly, the ratios of autofluorescence (keratin and collagen) expressed in the epithelium and the sub-epithelium did not distinguish pre-cancer stages well in epifluorescence (Fig. 4a). The features however distinguished the pre-cancer well with MUSI-tAF (Fig. 4b–e). A detailed comparison between epifluorescence and MUSI-tAF corresponding to Fig. 4b–e is shown in Supplementary Fig. S9. This implies that the relative expressions of collagen and keratin as derived by MUSI-tAF can be used to stage the disease label-free. We also used variance as a measure of tissue density variations in the layers and noted layer-wise measurements to delineate disease stages (Fig. 4f–k). This was expected since fibrosis leads to the accumulation of matrix proteins during OSF-linked matrix remodeling and makes the tissue more homogeneous^36^. In conclusion, we find that MUSI-tAF has the potential to delineate the different stages of OSF development. Widespread clinical studies on OSF, as well as other fibrotic diseases through newer clinically relevant MUSI-tAF features, can be explored in the future.

**Figure 5:**
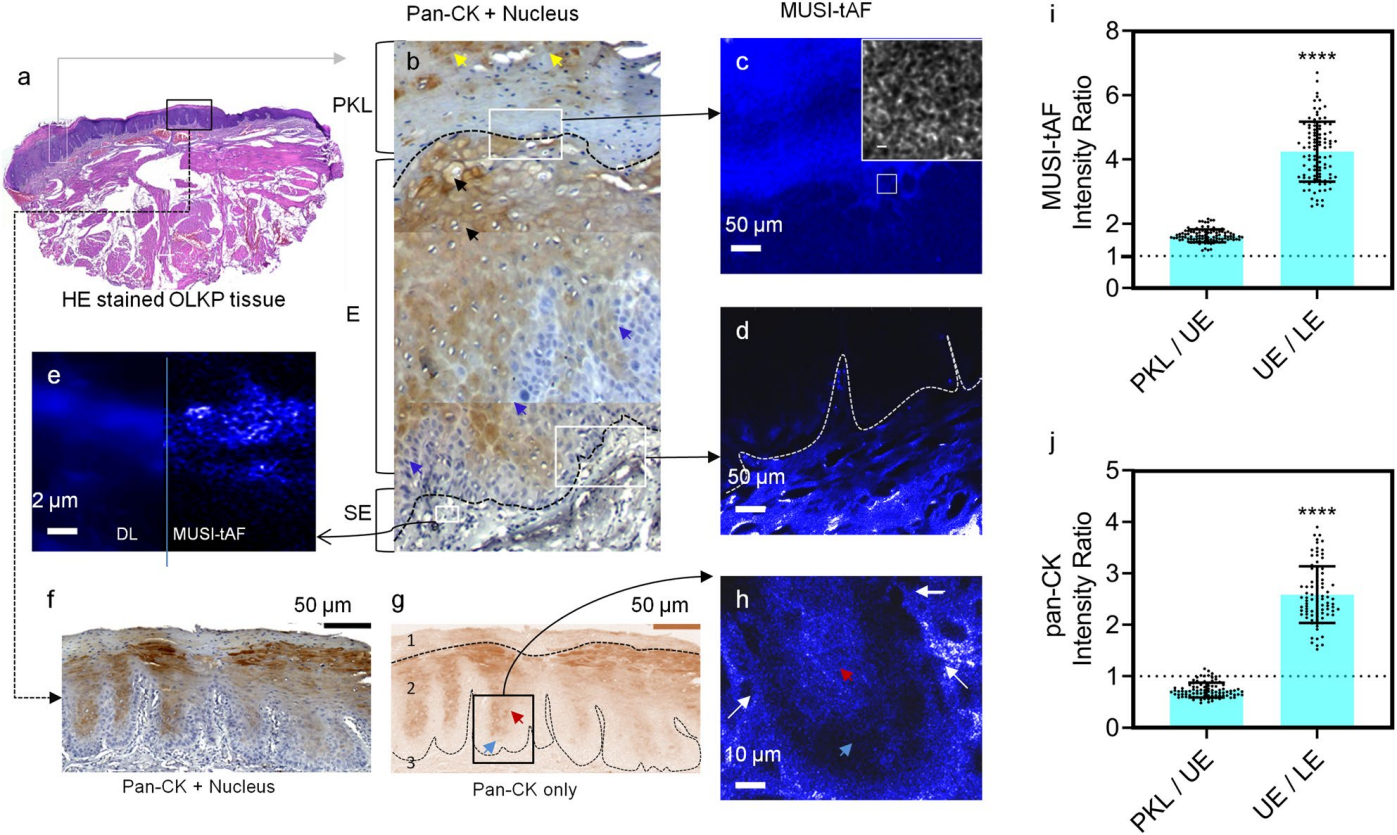
MUSI-tAF in detecting keratin-associated human oral leukoplakia. (**a**) Histology of tissue with leukoplakia, (**b**) pan-CK (keratin) stained region of interest to show the distribution of cytokeratin in the tissue, (**c**,**d**) are MUSI-tAF images showing different regions in the tissue. (**e**) Side-by-side view of epifluorescence and super-resolved image, (**f**) another region showing pan-CK distribution, (**g**) extracted image after color deconvolution containing only keratin density information, (**h**) is the region taken from the papillary region showing the distribution of keratin with MUSI-tAF, (**i**,**j**) Intensities of MUSI-tAF and keratin stain showing similar ratios of intensities. N = 100 per group (patient samples = 5 $\times$ ROI = 20), All plots are represented as $\mu$ ± SD, ****p $\le$ 0.0001. For statistical analysis, we used a two-tailed t-test (95% confidence interval).

### MUSI-tAF overcomes problems due to epitope masking

Oral leukoplakia (OLKP) is globally the most prevalent precancer of the mouth^37^. Abnormal deposition and condensation of keratin over the mucosal epithelium is a marker of OLKP, resulting in the para-keratinized plaque layer (PKL) (Fig. 5a,b). However, our results from keratin immunostaining (Fig. 5b) and prior literature^12,27,28^ suggest that labeling in highly condensed areas like the para-keratinized layer is inconsistent even with keratin-specific antibodies. The inconsistent labeling is due to modification of the binding site on keratin protein or its condensation, causing the masking of keratin epitopes, rendering it unavailable for labeling. The immunostained OLKP tissue shows almost half the occurrence of keratin found in epithelium-proper due to incomplete labeling in the para-keratinized layer (Fig. 5j), which contrarily is heavily keratin-dense^38^. As labeling artifacts do not limit MUSI-tAF, we observed the para-keratinized layer to have a 2-fold higher keratin expression than epithelium proper (Fig. 5c,i).

In the epithelium (specifically epithelial papilla), the basal and suprabasal layers (lower epithelium LE) are keratin deficient while the epithelium proper (upper epithelium UE) in OLKP is keratin-rich (Fig. 5b,f,g). Since no epitope masking hinders keratin labeling in the region, this observation matched MUSI-tAF and keratin labeling (Fig. 5h,i,j). Further, we observe a characteristic bundling of subepithelium in the MUSI-tAF of OLKP tissue (Fig. 5e) otherwise not seen in OSF or OSFD.

The differences in the relative expressions of epithelial keratin and sub-epithelial collagen are prominent for the dysplastic case of OLKP (Fig. 5d) but inconspicuous for OSFD (Fig. 3k,l). Therefore, in addition to identifying precancer stages, the MUSI-tAF can be used to delineate the type of precancer occurring in the same anatomy based on density variations of matrix proteins.

**Figure 6:**
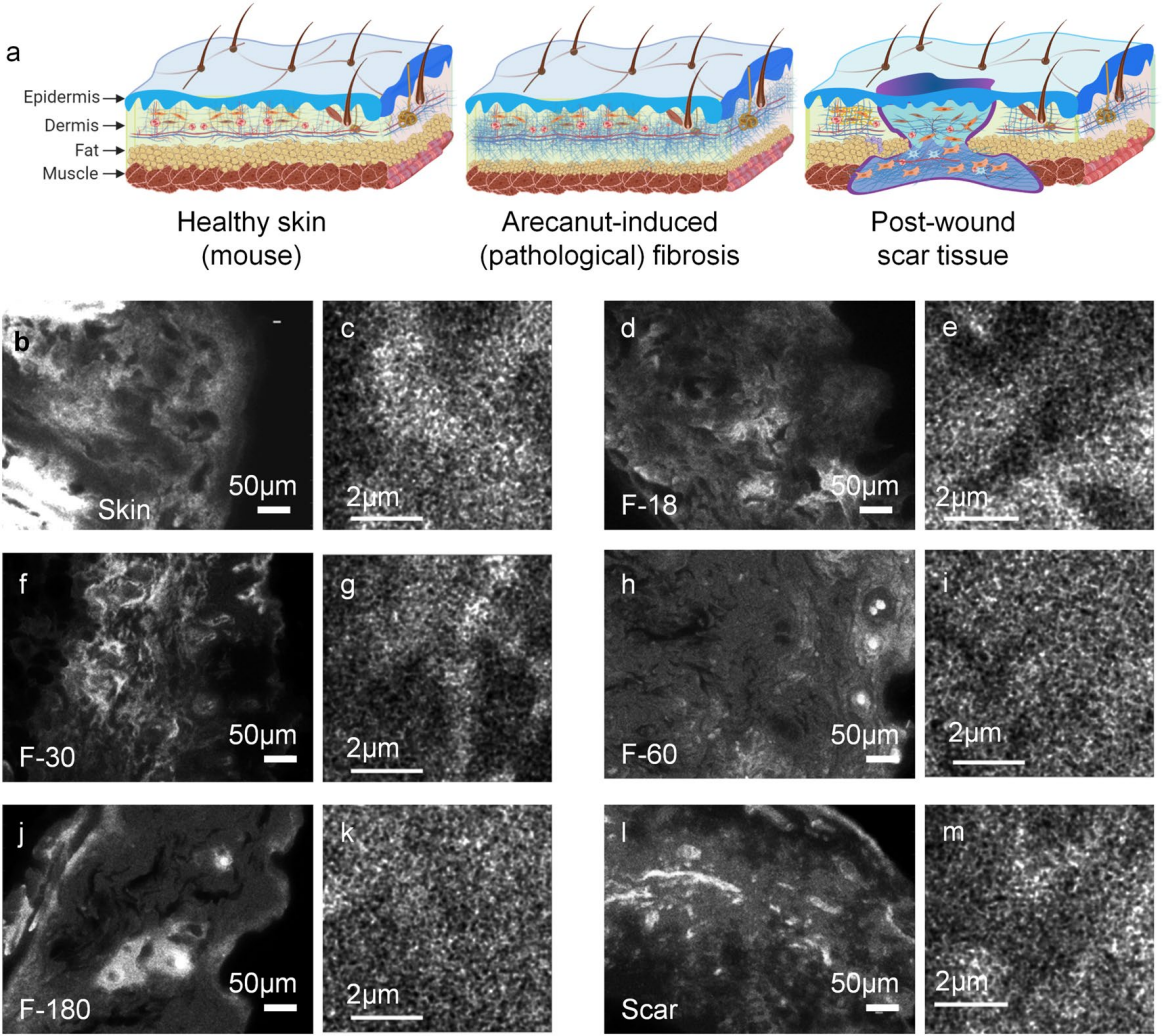
MUSI-tAF for wound healing scars and pathological fibrosis in mouse skin. (**a**) A pictorial representation of mice skin and changes it undergoes during arecanut-induced pathological fibrosis and post-wound scar tissue. A broad and small field of MUSI-tAF from the same sample for (**b**,**c**) healthy skin. MUSI-tAF of pathological fibrosis by arecanut extract (ANE) after application for (**d**,**e**) 18 days, (**f**,**g**) 30 days, (**h**,**i**) 60 days, and (**j**,**k**) 180 days. (**l**,**m**) broad and small MUSI-tAF field views of scar tissue after 60 days of healing of a full-thickness skin wound. All experiments are performed on dorsal mice skin.

### MUSI-tAF in pathological fibrosis and post-wound scarring

We explored the applicability of MUSI-tAF in (i) pathological fibrosis induced by topical application of arecanut extract on mouse dorsal skin and (ii) non-pathological fibrosis in normal wound scars on mouse dorsal skin (Fig. 6a). We chose arecanut extract (ANE) as the fibrosis-inducing agent in mouse skin as it is the key cause of the precancer-OSF in humans. ANE-induced fibrosis on mouse skin is thus used as a pre-clinical model of OSF^32,39–41^. We applied ANE for 180 days to mimic pathological fibrosis and study its progression using MUSI-tAF. We also applied MUSI-tAF on healed scars which is a non-pathological manifestation of fibrosis in the body as an end product of natural wound healing in mammals.

**Figure 7:**
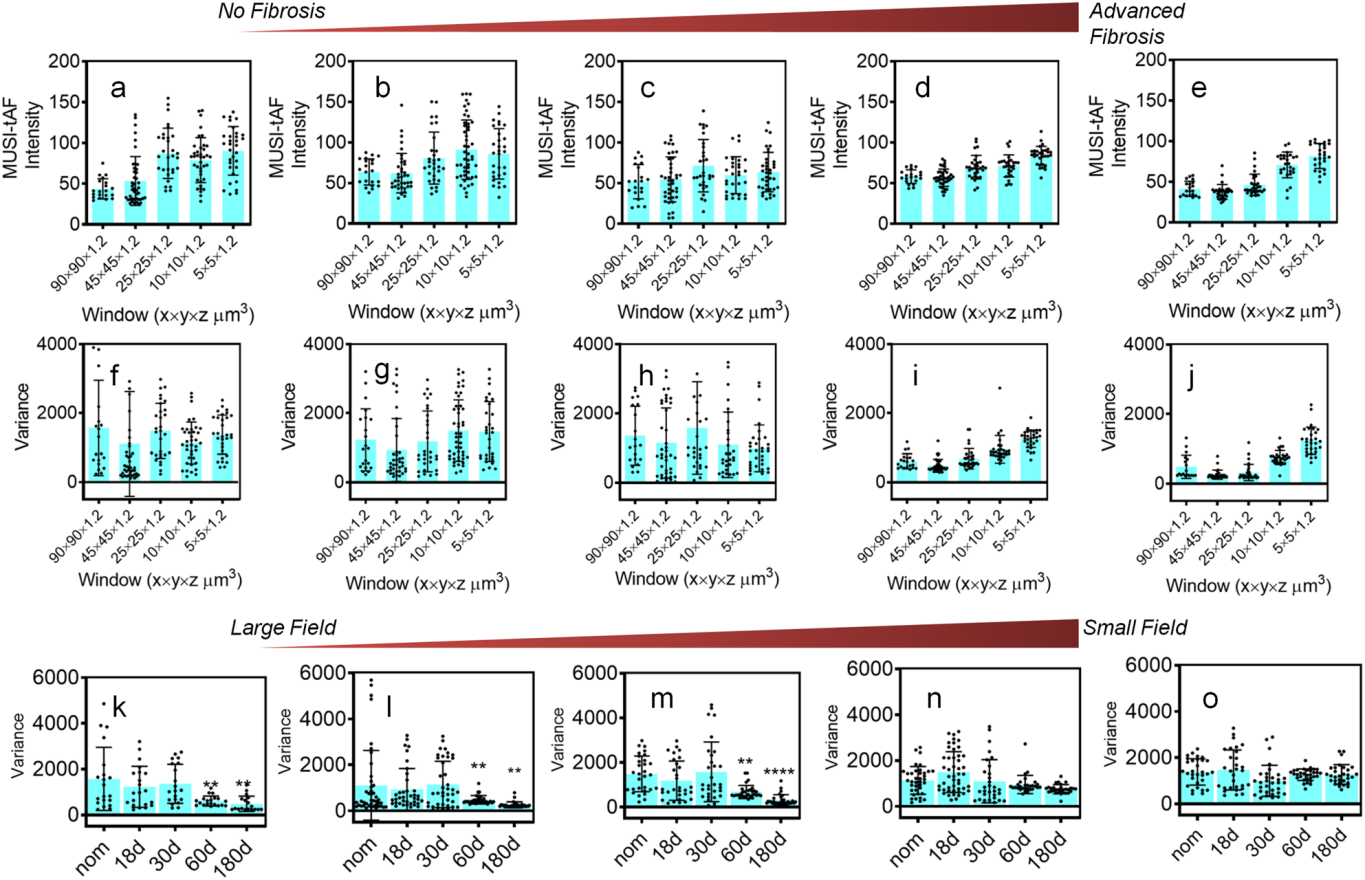
Multi-scale evaluation of pathological fibrosis and wound scars with MUSI-tAF in mouse skin. MUSI-tAF measurement of (**a**–**e**) intensities and (**f**–**j**) variance in different window sizes; (**a**,**f**) healthy skin, (**b**,**g**) 18-day fibrosis, (**c**,**h**) 30-day fibrosis (**d**,**i**) 60-day fibrosis, and (**e**,**j**) 180-day fibrosis. N = 20–40 (N = 4 animals $\times$ 10 ROI) per group. (**k**–**o**) Shows variance measurements between fibrotic stages, with window sizes (**k**) $90\times 90\times 1.2\upmu \textrm{m}^3$, (**l**) $45\times 45\times 1.2\upmu \textrm{m}^3$, (**m**) $25\times 25\times 1.2\upmu \textrm{m}^3$, (**n**) $10\times 10\times 1.2\upmu \textrm{m}^3$, (**o**) $5\times 5\times 1.2\upmu \textrm{m}^3$. N = 20 (N = 4 animals $\times$ 5 ROI) per group. All plots (**a**–**o**) are represented as mean ($\mu$) ± standard deviation (SD) using error bars. Significant differences are marked with * for *p* $\le$ 0.05, ** for *p* $\le$ 0.01, *** for *p* $\le$ 0.001, **** for *p* $\le$ 0.0001. For statistical analysis, we used one-way ANOVA (95% confidence interval). Only the groups showing significant differences are labelled. The non-significant differences are not labeled. The statistical comparisons are made only with respect to the controls (first column). Significant changes were found in images (**k**) to (**m**).

We observed that the density variations within the skin dermis gradually reduce with the progressive pathological fibrosis in all scales (Fig. 6b–k) (Supplementary Fig. S8a–t). To quantify this, we performed a multi-scale analysis of matrix variation from a broad field ROI (90 $\times$ 90 $\times$ 1.2 $\upmu \textrm{m}^3$) to a minimal field ROI (5 $\times$ 5 $\times$ 1.2 $\upmu \textrm{m}^3$) (Supplementary Fig. S8) and found that the variation within the ROIs (Fig. 7a–e) and between multiple such ROIs (Fig. 7f–j) decrease with pathological fibrosis. The finding suggests pathological fibrosis causes collagen density to become homogenous within a given volumetric field, especially in the late stages of fibrosis (60 days and beyond). A similar observation was found previously in OSF sub-epithelial tissue (Fig. 3g). Further broader ROIs (Fig. 7k–m) produced better delineation of fibrotic progression over smaller ROIs (Fig. 7n,o) based on collagen variations.

## Discussion

MUSI-tAF achieves label-free nanoscopy of dense structures such as collagen and keratin and retains clinically relevant scales by using a low magnification objective that supports large lateral (448 $\times$ 335 $\upmu$m^2^) and axial field (1.2 $\upmu$m). Importantly, MUSI-tAF improves the visualization and density representation of collagen fibrils at a macro scale (across several hundreds of microns) through improved lateral resolution and rejection of the out-of-focus light. Such utilization of super-resolution for macro scale clinically relevant analysis has not been reported before, to the best of our knowledge. MUSI-tAF, therefore, presents an exciting new tool for label-free histology and extends the utility of conventional nanoscopy beyond nanoscale biological investigations to routine and clinically practical pathological investigations.

FFPE sections are prone to formalin-associated autofluorescence that can interfere with autofluorescence signals from collagen. In our work, we addressed this by performing epitope retrieval in the sample pre-processing stage which breaks these autofluorescence-inducing crosslinks^42^ (See Supplementary Fig. S2). Another concern is that water content in tissues can attenuate the autofluorescence signals. Increased exposure time can compensate for this but introduce other problems. These include quenching due to longer imaging time and reduced fluctuations in autofluorescence due to averaging effect of long exposure. We avoided these problems by performing a two-step dehydration of the tissue as explained in the methods. One of the currently unresolved problems of autofluorescence is the lack of specificity in a heterogeneous system such as tissue. Immunofluorescence labeling is a natural choice for studying protein-specific distributions in such cases. However, in our study, we found that autofluorescence provides a better structural representation of collagen fibrils than immunofluorescence (Supplementary Figs. S4, S5). Moreover, in our study involving oral and skin tissues, the exclusive presence of collagen and keratin in sub-epithelial and epithelial layers helped resolve their autofluorescence spectral overlap^33^. Nonetheless, the challenge of demarcating the sub-epithelial and epithelial layers still remains. In our study, this demarcation was done by two oncopathologists based on their domain knowledge. Therefore, here this distinction is only as subjective as the existing clinical knowledge. However, with MUSI-tAF images, more features with possibly better classification ability may be investigated and rigorous regression analysis may be incorporated in the future.

The spectral overlap of autofluorescence of keratin and collagen^33^ helped us to image the parallel expression levels of the two proteins separated by the basement membrane^43^. Our results in Fig. 3 showed a correlation between the changes in collagen and keratin expressions, which hints at cross-talk between sub-epithelium and epithelium layers. A possible explanation of this cross-talk is a two-way communication between the two proteins. The increased expression of basal/suprabasal keratin in epithelium with fibrosis impacts collagen accumulation in the sub-epithelium. On the other hand, increased mechanical stiffness due to collagen accumulation in sub-epithelium can affect cellular behaviour^44^ in the epithelium, resulting in altered keratin expression in the epithelium. OSF is a disease of collagen abnormality in the sub-epithelium that progresses into the cancer of epithelium^45^. Potentially, the cross-talk between collagen and keratin has specific clinical relevance^46^ and our studies have further confirmed such an event. Thus more detailed investigation on this need to be conducted in the future.

Due to a shared etiological factor (arecanut), fibrosis in mouse skin is a pre-clinical disease model of human OSF^32^. MUSI-tAF images demonstrated simultaneous loss of variation of density and collagen fibril diameters in mouse skin with pathological fibrosis. We compared scars after wound healing and found matrix features that are different from healthy tissue as well as pathological fibrosis. Using density and collagen fibril dimensions, we distinguished pathological fibrosis (arecanut-induced fibrosis) from a non-pathological one (wound scar). Further, our MUSI-tAF results for distinguishing the scar compartments are in agreement with recent studies^47^ that discuss their distinct composition, mechanical properties, and relevance to their stress-management adaptation.

Multiple signal classification algorithm itself has been applied to fluorescence, and therefore its own applicability is much wider. Here we focus on the autofluorescence aspects of dense collagen and the value additions it can bring to diagnosis. Before MUSI-tAF, super-resolution techniques for autofluorescence from dense tissue collagen with a clinically relevant field were unavailable. MUSI-tAF resolves this problem, provides label-free super-resolution, and demonstrates the relevance of super-resolution for macro-scale density features for histology. This has enabled both clinical and pre-clinical studies related to fibrosis. MUSI-tAF itself is a generalizable concept, which can be of direct use for any autofluorescence protein whose nanoscale structure has clinical relevance on a macroscopic scale. Further, the methods developed in this work apply to all FFPE samples and hence can be used widely as a value-added tool in addition to routine histopathological and molecular pathology examinations. In addition, MUSI-tAF is amenable to correlative label-free and labeled microscopy techniques within the wide-field microscopy umbrella. Therefore, more powerful clinical applications of a wide variety are likely to emerge in the future. Lastly, we note the wide applicability of our work in the precise context of collagen. Collagen abnormality is the primary marker of fibrosis. Therefore, our work has a wide impact on all conditions related to fibrosis. Its significance can be estimated by the fact that fibrosis affects all vital organs of the body and is responsible for 45% of the deaths in industrialized nations^48^.

Super-resolution microscopy has been always used in the context of seeking smaller and smaller structures. This study highlights that super-resolution is able to quantify features in the macro scale i.e. density in this case, a rather simple effect of the intended super-resolution. Besides density, other potential features of interest are the fiber dimensions and edge detection at the interface of the epithelium and sub-epithelium. For example, in oral submucous fibrosis, a significant change is observed in the basement membrane that gets flatter with the progression and eventually breaks in cancer due to epithelial tissue invasion. Applying methods such as edge detection can help in identifying the epithelial islands and therefore the extent of the spread of the cancer. The MUSI-tAF images can also be integrated with automated classification pipelines for making quick clinically supportive decisions. The key features like the density ratio between different layers of the oral mucosa, edges of the basement membrane, contours of invaded tissue islands, density measurements of proteins, and variance of matrix proteins can be used and incorporated into machine learning algorithms, holding the potential for classifying early stages of oral cancer. Further, other simpler optical microscopy methods like deconvolution microscopy can also alternatively attain a similar outcome. The deblurring algorithms like deconvolution are computationally economical. However, a detailed study is required to evaluate its potential with autofluorescence in clinical samples such as signal-to-background ratio and additive noise from multiple z-planes. Further, features whose point spread functions overlap in particular z planes may be sharpened in planes where they do not belong causing the apparent position of features to be altered. This problem is particularly severe when deblurring single two-dimensional images because they often contain diffraction rings or light from other structures that will then be sharpened as if they were in that focal plane. Other deblurring algorithms like Wiener filter, Richardson Lucy, blind deconvolution remove blurred signals and thus reduce overall signal levels besides other constraints that need to be optimized for fixed tissues but can potentially achieve higher contrast by deblurring.

While using the MUSICAL algorithm, it is important to ensure that the signal-to-background ratio is at least 3 or above. This allows for the algorithm to estimate the noise and out-of-focus light more accurately and effectively remove them. To achieve this, the sample processing steps before imaging must be followed as described. It is also important to make sure exposure time is not too short so images will get noisy and exposure time is not too long so fluctuations from the signals are averaged out in time. We used a stack of 500 images with 50 ms exposure time for each image to achieve MUSI-tAF with the given sample processing conditions. Although this can be achieved with fewer frames as low as 50, we recommend 500 frames as we observed no significant loss of autofluorescence signal in this duration of image acquisition.

## Conclusion

We elaborate MUSI-tAF-a label-free super-resolution method that obtains nanostructural details and density variation across a broad field of dense tissue matrix proteins. MUSI-tAF uses low magnification objectives, protein autofluorescence, and the MUSICAL algorithm to resolve collagen and keratin fibril structures and can be used to detect minute density changes between tissues undergoing fibrotic maladaptations. We applied MUSI-tAF to extract pathologically relevant features in pre-cancer staging, pathological fibrosis, and post-wound scars. Since several diseases are associated with tissue matrix integrity and distribution, the translation of MUSI-tAF to histopathology can add value to the arsenal of existing pathological practices for label-free molecular diagnosis.

### Supplementary Information

Supplementary Information.

## Data Availability

All data can be availed upon request to the lead author Biswajoy Ghosh. The link to the multiple signal classification algorithm and its application is provided in the Methods section.
