# Supplementary material for: Molecular histopathology of matrix proteins through autofluorescence super-resolution microscopy
Source: Sci Rep. 2024 May 8;14:10524. doi: 10.1038/s41598-024-61178-0 (PMC11078950; doi:10.1038/s41598-024-61178-0)
Supplement: Supplementary file 1 — Supplementary Information. [file 41598_2024_61178_MOESM1_ESM.pdf]

Supplementary to: Molecular Histopathology of Matrix Proteins through Autofluorescence Super-resolution Microscopy

Biswajoy Ghosh[1,3,*], Jyotirmoy Chatterjee[1], Ranjan Rashmi Paul[2], Sebastian AcuÑa[3], Pooja Lahiri[1], Mousumi Pal[2], Pabitra Mitra[1], and Krishna Agarwal[3,*]

[1]Indian Institute of Technology Kharagpur, West Bengal, 721302, India
[2]Guru Nanak Institute of Dental Sciences and Research, Kolkata, West Bengal, 700114, India
[3]UiT - The Arctic University of Norway, Tromsø, 9019, Norway
[*]biswajoy.ghosh@uit.no
[*]krishna.agarwal@uit.no

The document contains Supplementary Table S1 and S2, Supplementary Figures S1-S9 and Supplementary Notes 1-4

Supplementary Tables

Source	Group	Number of samples	Number of image stack per sample	Number of ROI for intensity measurements
Human patients	NOM	5	3	30
	OSF	5	3	30
	OSFD	5	3	30
	OSCC	5	3	30
	OLKP	5	3	30
		25 Total*	25 x 3 = 75 Total image stacks	75 x 30 = 2250 Total ROI's
Mouse	Healthy skin	4	3	30
	18-day fibrosis	4	3	30
	30-day fibrosis	4	3	30
	60-day fibrosis	4	3	30
	180-day fibrosis	4	3	30
		20 Total	20 x 3 = 60 Total image stacks	60 x 30 = 1800 Total ROI's

Table S1. Overview of the numbers of samples, images and ROI's used in the study. *The pathologists select these 25 samples according to their exclusion criteria, where they choose samples that do not have other complications.

Disease	Metrics	Explanation and Clinical relevance
Human Oral Submucous Fibrosis	SE/E	The overall collagen density change in sub-epithelium with respect to keratin density change in Epithelium. Provides an overview of how the density of these two molecules is effected when the pre-cancer progresses to cancer.
	UE/LE	The density change of keratin in upper epithelium relative to lower epithelium. The disease originates in lower epithelium with high keratin expression relative to upper epithelium. Lower value denotes normal and the value increases with disease progression.
	USE/LE	The density of collagen in upper sub-epithelium relative to keratin in lower epithelium. These two sub-layers are closely interacting with each-other and is at the junction of the site of invasion. This is an important metric to evaluate the relative collage keratin interaction in early onset of pre-cancer and therefore, a valuable early-marker.
	LSE/USE	The density change of collagen in upper sub-epithelium relative to lower sub-epithelium. This represents papillary and reticular regions of sub-epithelium which increasingly gets indistinguishable with advancement of oral sub-mucous fibrosis.
Human Oral Leukoplakia	PKL/UE	The density of keratin in para-keratinized layer relative to upper epithelium. The density of para-keratinized layer at this junction indicates progression of the disease. The keratin in the PKL layer are highly compressed and difficult to effectively stain due to antigen blocking, but are easily discernible with auto-fluorescence.
	UE/LE	The density of keratin in upper epithelium relative to lower epithelium.

Table S2. Overview of the clinical metrics used in the study. This table refers to the metrics used in Fig 4 and Fig 5 of the article. For mouse dermal fibrosis, all intensity measurements are taken relative to the *stratum corneum* of the skin (outer most exposed layer of skin) in the Fig 7 of the article .

Supplementary Figures

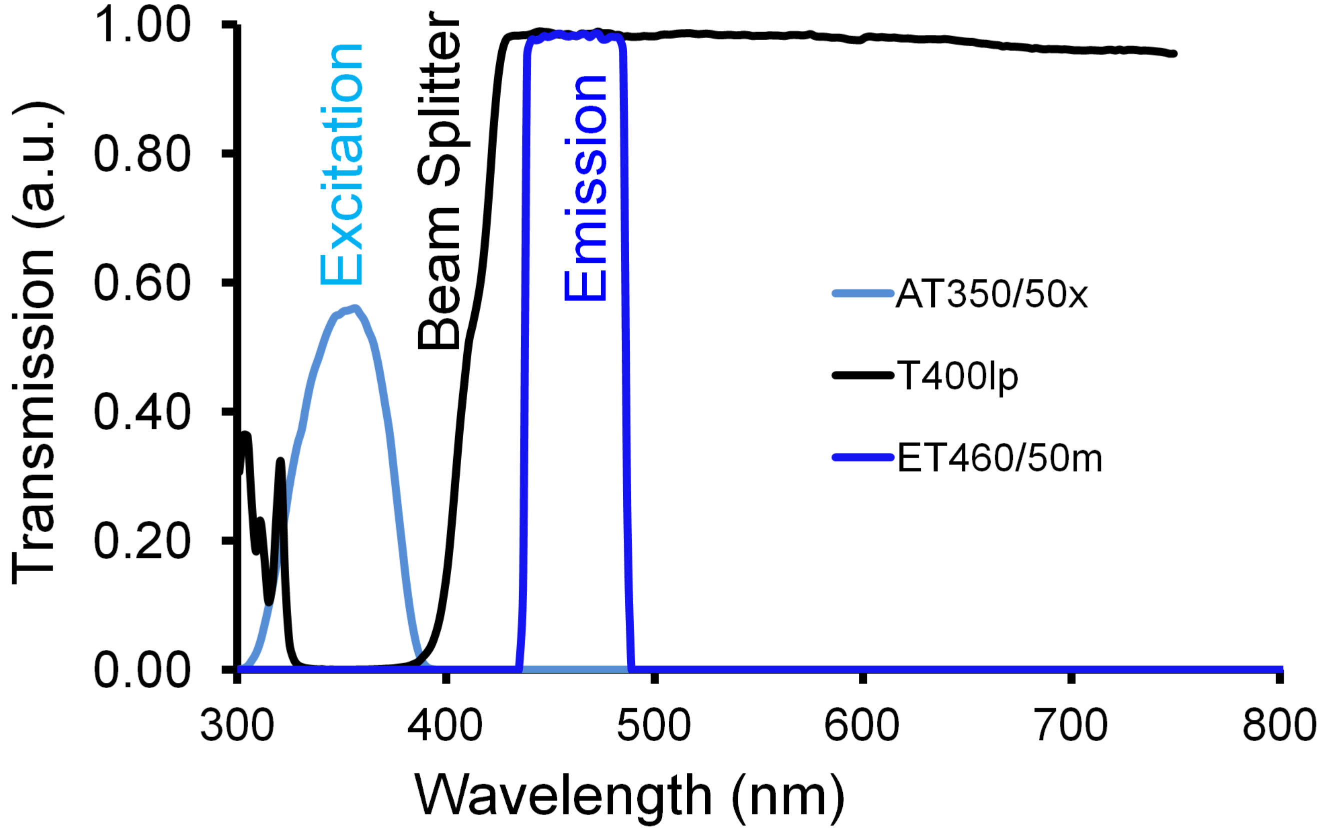

Figure S1. Fluorescence spectra for MUSI-tAF of matrix proteins. Figure shows the fluorescence filter's spectral characteristics for capturing the matrix proteins—collagen and keratin from tissue sections. The optical filter-set of epifluorescence microscope uses a $\overline{\lambda_{ex}}$= 350 nm, $\Delta\lambda$=100 nm for excitation and a $\overline{\lambda_{em}}$= 460 nm, $\Delta\lambda$=100 nm. A long pass filter beyond 400 nm is used as a beam splitter.

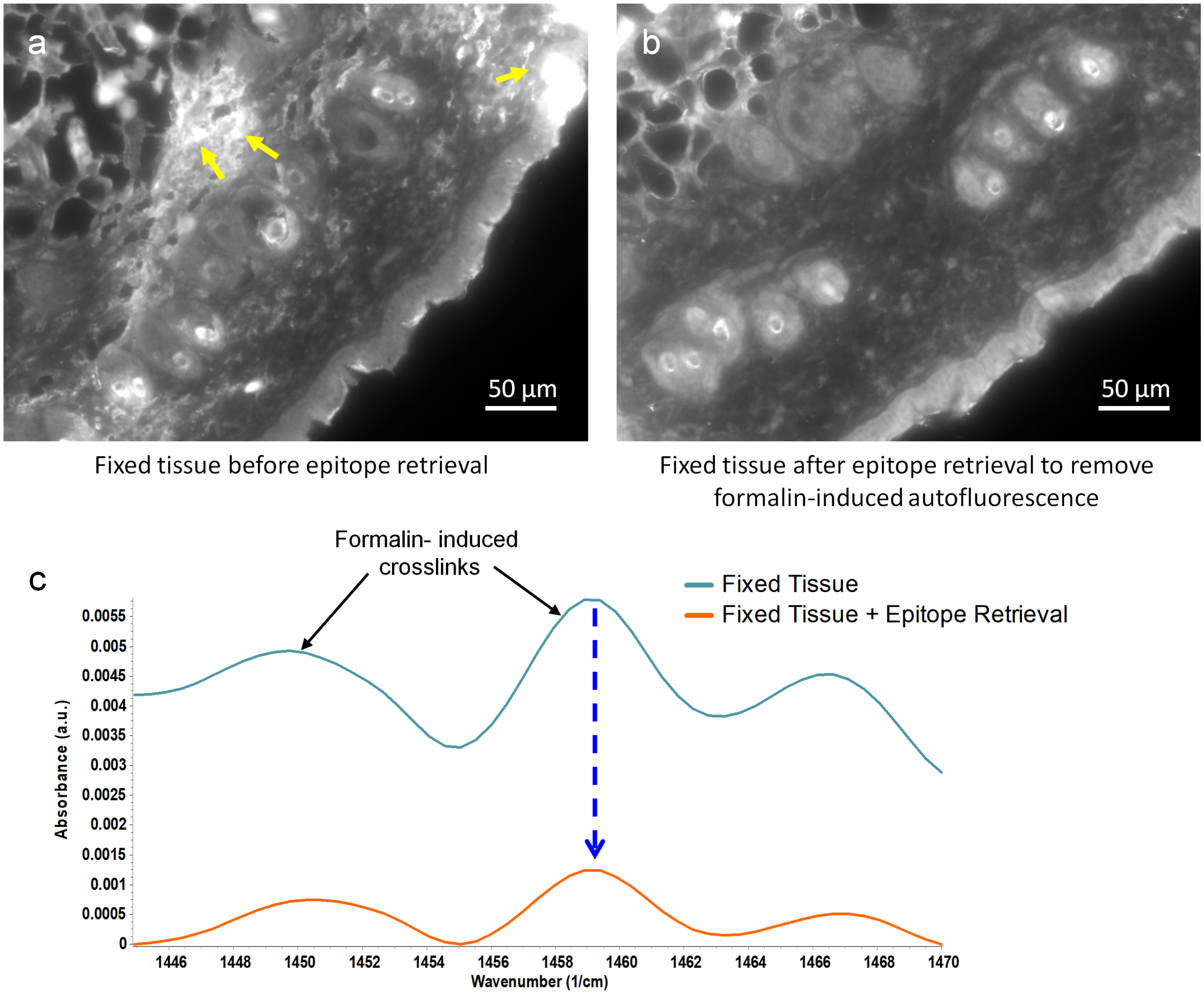

Figure S2. Effect of epitope retrieval in reducing the formalin-induced fluorescence (a,b) are the autofluorescence images of the same mouse skin tissue section taken before and after heat-induced epitope retrieval (same tissue section, image captured at similar location before and after processing). The yellow arrows show the regions with non-uniform fluorescence before epitope retrieval. (c) FTIR spectra of the adjacent mouse skin tissue sections with and without epitope retrieval. The spectra shows that the peaks of methylene bridges (formalin-derived crosslinks that cause unspecific autofluorescence) are substantially reduced upon epitope retrieval.

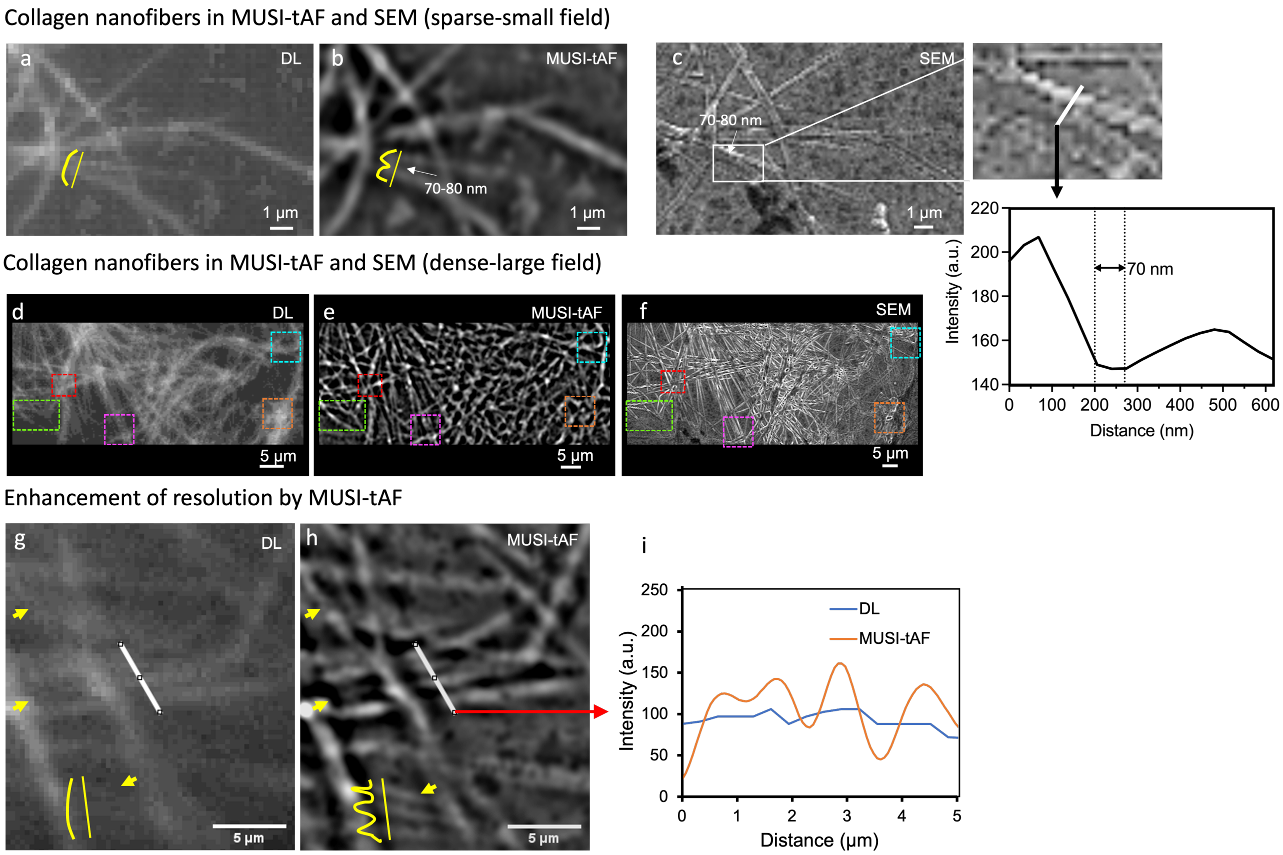

Figure S3. Benchmarking MUSI-tAF super-resolution in collagen nanofibers. A region of thinly deposited collagen-I under (a) diffraction-limited (mag 20×), (b) MUSI-tAF, and (c) SEM showing that MUSI-tAF images collagen nanofibers and can distinguish collagen spaced 70-80 nm apart (edge to edge). A denser region of rat tail collagen-I fibers under (d) diffraction-limited, (e) MUSI-tAF, and (f) SEM showing matching regions. The (g) diffraction-limited and (h) MUSI-tAF images of the same region demonstrate enhancement of resolution in closely spaced collagen fibers (yellow arrows). The profiles along the white line is shown in (i) demonstrating that MUSI-tAF super-resolves a nearly flat profile in DL imaging of four closely spaced collagen bundles. The colored boxes in the images (d,e,f) highlight the regions of the fiber structure visualizing the matching between the DL, MUSI-tAF and the SEM images respectively. The boxes of the same color show the matching region.

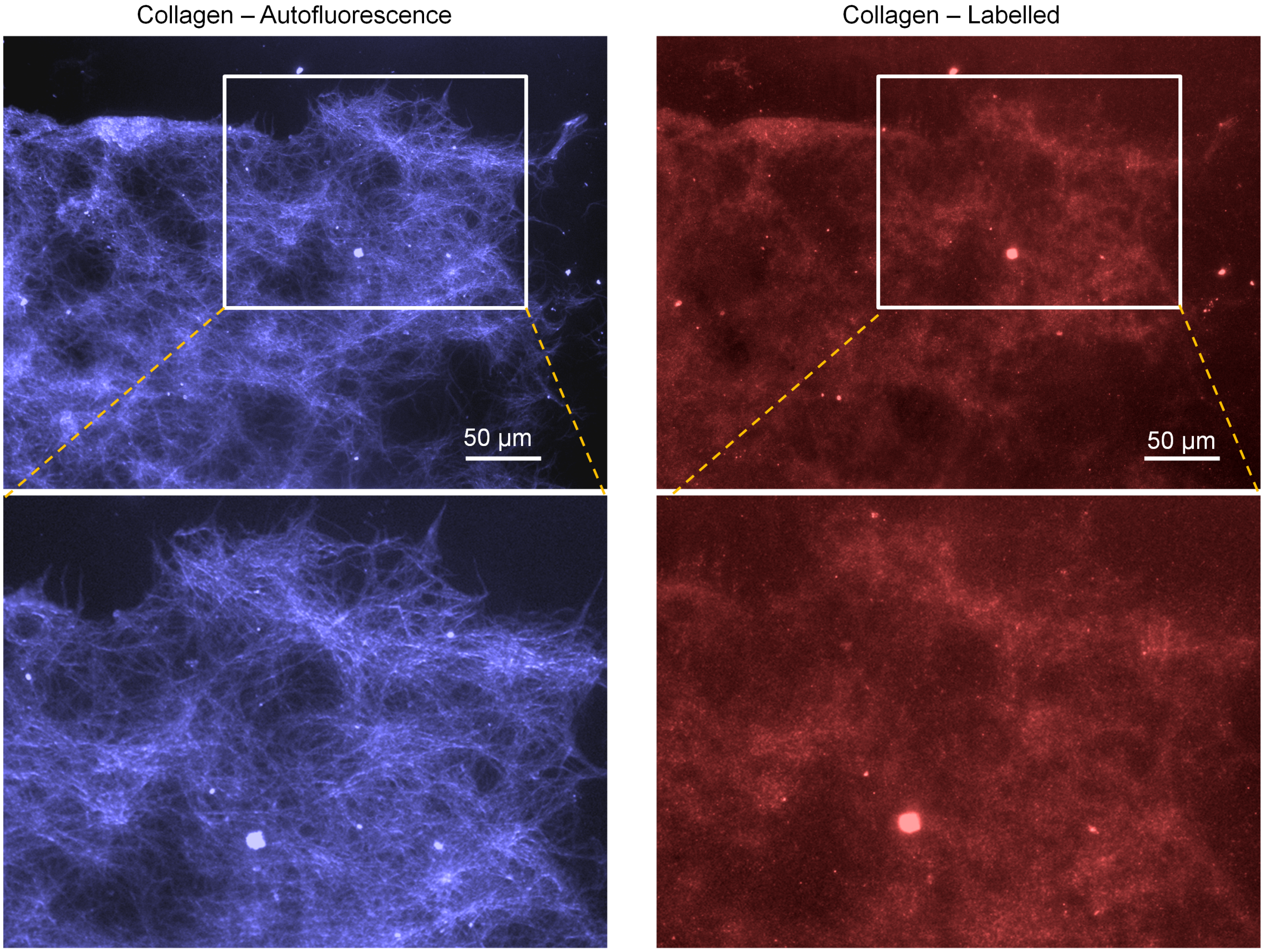

Figure S4. **Collagen autofluorescence provides a faithful distribution and localization than its labeled microscopy.** The left column shows collagen autofluorescence (blue channel) and the right column is the collagen labeling (red channel) taken from the same region. The zoomed images show that although labeling can provide a visual correspondence of collagen density it has poor localization of fibers. The images were acquired at 20× magnification, 0.8 NA.

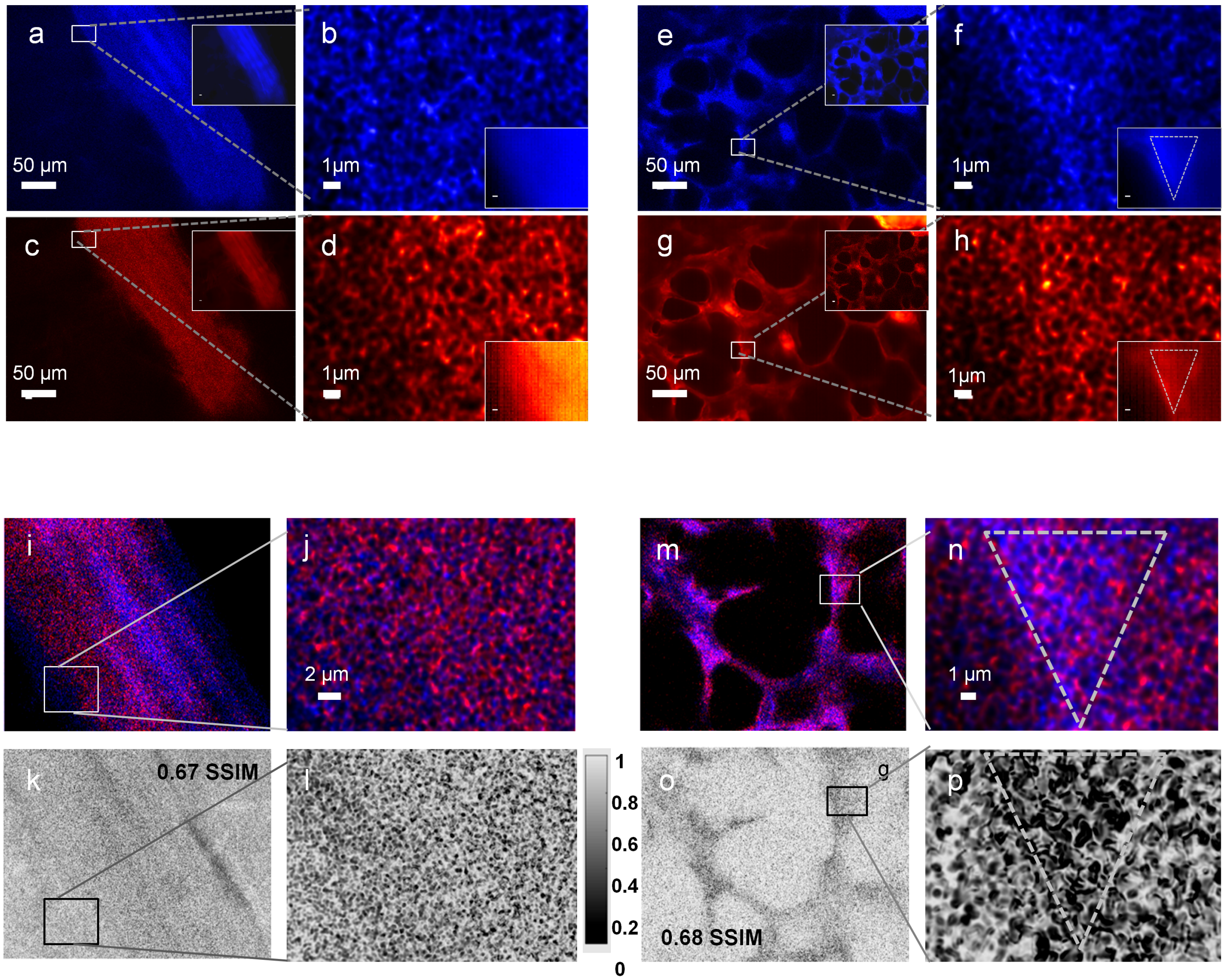

Figure S5. **Comparing collagen nanostructures in MUSI-tAF autofluorescence and labelled fluorescence.** MUSI-tAF images of (a-d) purified collagen-I and (e-h) mouse skin tissue sections. Images derived from (a,b,e,f) tissue autofluorescence (tAF) in blue emission, (c,d,g,h) same tissue immunolabeled with collagen-I and fluorescent probe having red emission. All insets show the corresponding source (diffraction-limited) images (20×, 0.8 NA objective). (a,c,e,g) are full-field super-resolved images and (b,d,f,h) are a small region illustrating super-resolved structures. Co-localization of structures of (i,j) pure collagen-I and (m,n)tissue from autofluorescence (blue) and labeled (red) fluorescence. Similarity maps of MUSICAL structures in the red and blue colors images in (i,j) are shown in (k,l) for Collagen-I, and similarly similarity maps of the structures in red and blue colored images in (m,n) are shown in (o,p) for the mouse skin tissue.

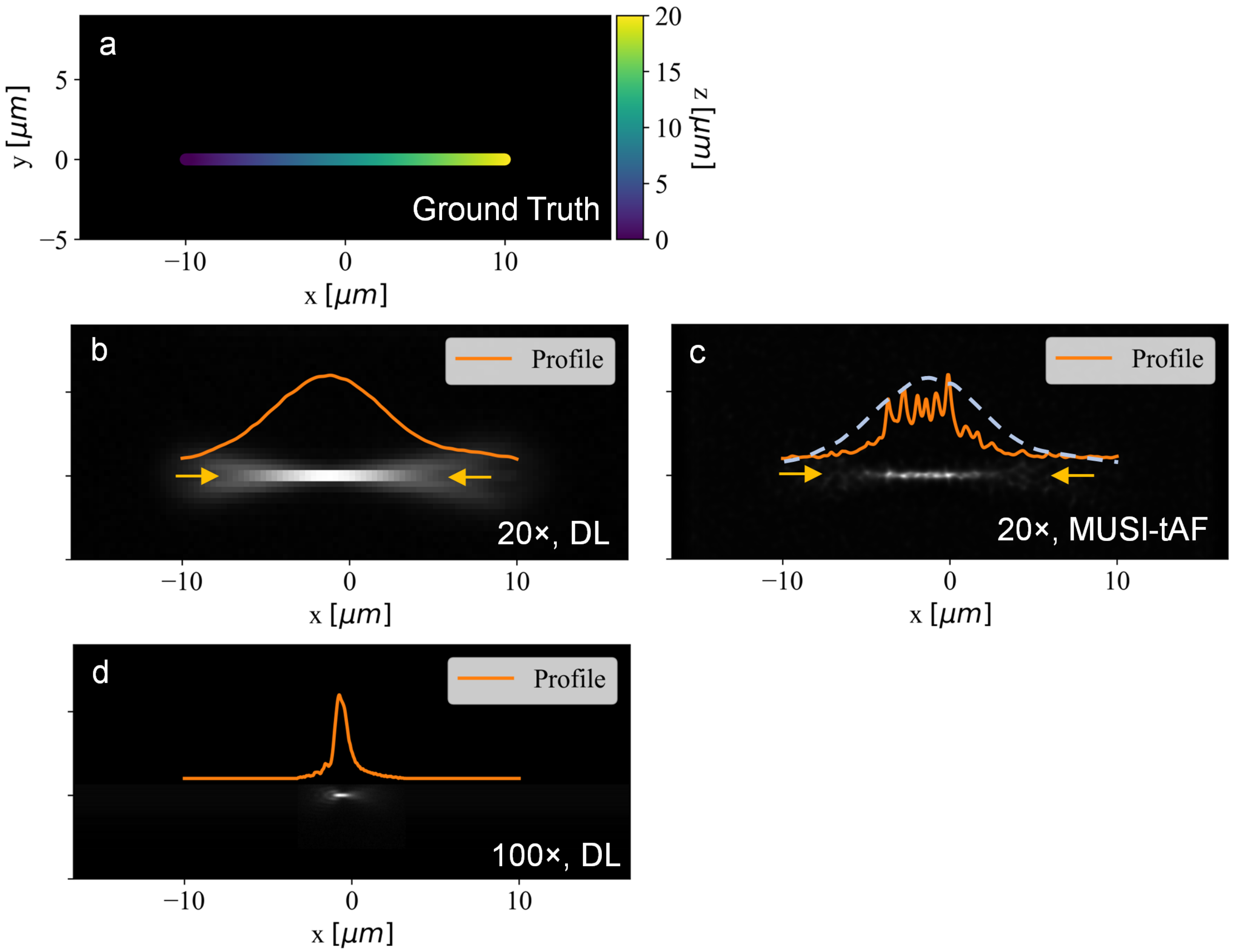

Figure S6. Simulated sample showing emitters placed along a straight line at a distance of 10 μm, showing the rejection of out-of-focus light property in MUSI-tAF. The imaging process was simulated using a 20× and 100× objective with Poisson noise. (a) The line passes through several planes at different z-positions and is the ground-truth. The color bar indicates the distance of the emitter and the coverslip. (b) 20× objective diffraction limited image over 100 frames created for the sample. The edges of the line (arrows) show the effect of the point-spread-function being wider at off-focus regions. (c) MUSI-tAF results for the 20× objective where the focal section is filtered from the off-focus emission. The dotted line is the profile of the corresponding diffraction limited line showing the region of rejection on the edges. (d) Profile plot of a 100× diffraction limited image.

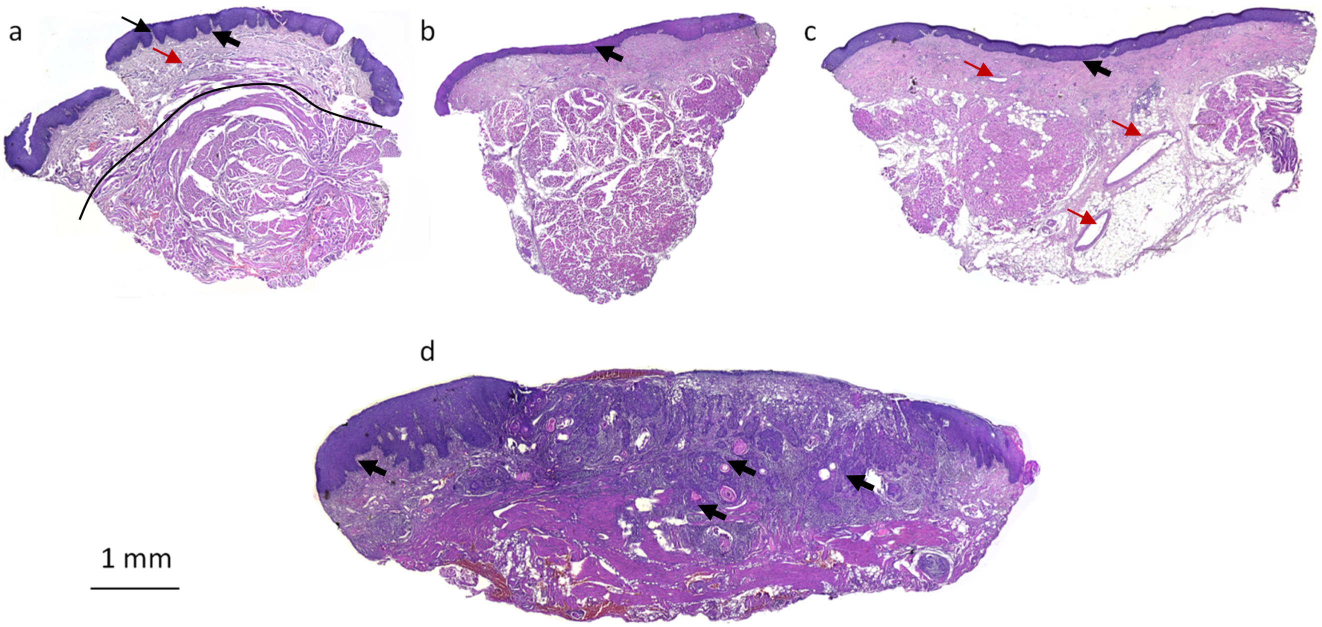

Figure S7. Histology of representative full oral tissues sections with varying levels of pathology associated with oral carcinoma. (a) Hematoxylin and eosin (HE) stain of the normal oral mucosa (NOM), the darker stained outer epithelium (black arrows), and sub-epithelium (red arrow). The region below the black line is majorly muscle tissues; the bold black arrow shows the epithelium's rete pegs, i.e., undulations at the base of the epithelial layer. (b) HE of oral submucous fibrosis (OSF) shows denser epithelium and flattened rete pegs (bold arrow). (c)HE of oral submucous fibrosis with dysplasia (OSFD),) with flattened rete pegs (bold arrow), dense sub-epithelium, and perivascular fibrosis (red arrows). (m) HE of oral squamous cell carcinoma (OSCC), with the epithelial basement membrane, lost continuity and invaded into subepithelium (bold arrows).

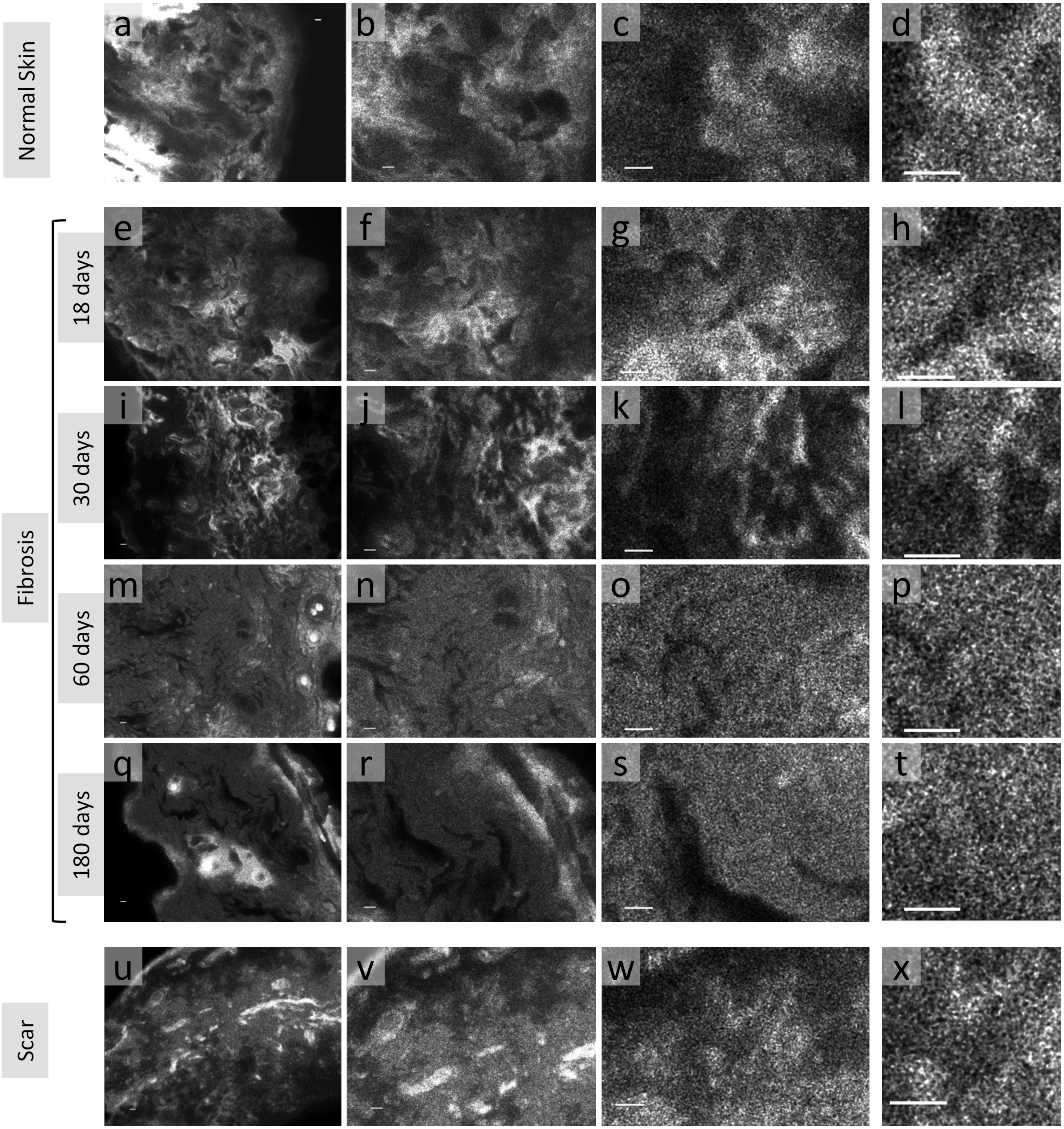

Figure S8. Multi-scale MUSI-tAF images of healthy and fibrotic skin tissues. (a-d) MUSI-tAF images of a healthy skin tissue visualized at image sizes of (a) 10000×13400 (full field of view), (b) 4000×5000, (c) 2000×3000, and (d) 1000×1000. The four image dimensions have been illustrated for progressive pathological fibrosis at (e-h) 18 days, (i-l) 30 days, (m-p) 60 days, and (q-t) 180 days; here, while 18 days and 30 days treatment are early fibrosis, 60 days and 180 days are advanced fibrosis. (u-t) images of scar tissue collected from 60 days of wound healing at the same dimensions as healthy skin. *scale bar*= $2\mu m$.

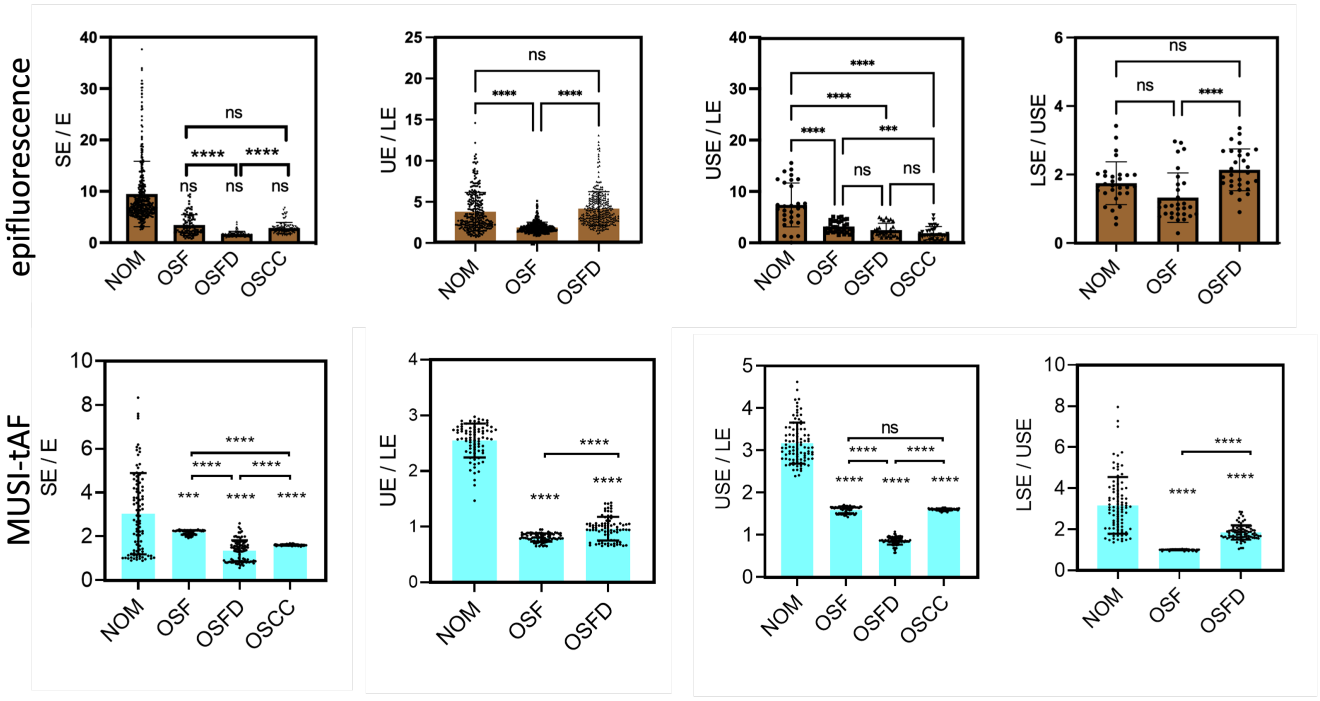

Figure S9. Comparison of epifluorescence and MUSI-tAF intensity ratios in different regions of the oral tissue. The figure highlights how MUSI-tAF can make better delination of disease stages compared to epifluorescence imaging of autofluorescent tissue matrix. This figure corresponds to Fig 4 (a-e) in the main manuscript.

Supplementary Notes

Note1: SEM is used for benchmarking as it confirms the structures present in the sample as well as proves that the rendered structures are not the result of artifacts. However, since SEM only images surface structures, it is important to use a thin section of collagen as a test object for one-to-one matching. Here, we deposited dense but thin collagen fibrils of monolayer thickness as a control sample (Fig S3). We demonstrated that MUSI-tAF structures resemble the collagen nanofibrils seen in SEM for both sparse (Fig S3 a-c) and dense regions (Fig S3 d-f) of the sample. This confirms that the rendered structures are not artifacts. Furthermore, we found that MUSI-tAF cannot resolve fibers that are overlapping (very dense matrix), however only when the spacing between the fibers is at least 70-80 nm apart edge to edge (see arrow in Fig S3 b, c), the two fibrils could be distinguished. The resolution of the system is therefore about 300 nm considering the peak-to-peak distance between adjacent fibrils. Thus for the blue emission, and the optics used (20×, 0.8 NA) the resolution achieved is close to the theoretical diffraction limit (Abbe's resolution limit of 575 nm). This implies that the method should be able to resolve close collagen fibers in not very dense samples, such as also shown in Fig S3 g-i.

Note2: To validate the structural reliability of MUSI-tAF, we considered matching the super-resolved autofluorescence results with that of collagen labels. However, the diffraction-limited images showed that the autofluorescence had higher structural reliability than labeled collagen (Supplementary Fig S4).

Note3: After comparing the super-resolution of the autofluorescence and labeled fluorescence we obtained a similarity score of 60-80% (Supplementary Note 1, Supplementary Fig S5). This relatively low similarity can be attributed to labeling inadequacies as shown in Supplementary Fig S4. Nonetheless, we do not discount the utility of fluorescence and immunolabeling; we only suggest that our solution may be more effective in clinical settings where immunostaining can be prohibitive.

Note4: Comparing super-resolved structures from autofluorescence and labeled fluorescence of collagen. We used purified collagen-I fibers and compared the super-resolved structures obtained from MUSI-tAF on autofluorescence (blue) (Fig S5 a,b) and MUSI-tAF on collagen-I specific labeling (red) (Fig S5 c,d). In full-frame images (Fig S5. a,c), although both autofluorescence and labeled images appear similar, we noted that the autofluorescence provided more prominent density variations of the bundles. Co-localization between diffraction-limited images of autofluorescence and labeled fluorescence presented a similarity of 96-98% (Supplementary Fig S3a). On the other hand, super-resolution on both autofluorescent and fluorescent-labeled collagen sample images exhibit 60-80% similarity, depending on the region (Fig S5 i-l). Since molecular specificity was not a problem for autofluorescence when imaging purified Collagen-I, the source of difference is the subjectivity of fluorophore labeling and the spatially shifted molecular localization in labeled fluorescence. A zoomed-in MUSI-tAF (Fig S5 b,d) autofluorescence shows a uniform density while in the labeled image, it presents a non-uniform distribution of collagen. Since we purely imaged collagen I from the collagen-I-only sample, the autofluorescence emitter-density information is more reliable than fluorescent labeling (Supplementary Fig S4), this mismatch is expected to translate into a low SSIM score between super-resolved structures derived from autofluorescence and labeled fluorescence. We also made a structural comparison between autofluorescence and labeled fluorescence in the collagen-rich animal skin connective tissue (Fig S51 e-h). The similarity between super-resolved images from autofluorescent and fluorescent-labeled tissues was 60-80%, the range is similar to that observed in purified collagen-I samples (Fig S5 m-p).
